# Condensin II drives large-scale folding and spatial partitioning of interphase chromosomes in *Drosophila* nuclei

**DOI:** 10.1371/journal.pgen.1007393

**Published:** 2018-07-12

**Authors:** Leah F. Rosin, Son C. Nguyen, Eric F. Joyce

**Affiliations:** Department of Genetics, Penn Epigenetics Institute, Perelman School of Medicine, University of Pennsylvania, Philadelphia, Pennsylvania, United States of America; Geisel School of Medicine at Dartmouth, UNITED STATES

## Abstract

Metazoan chromosomes are folded into discrete sub-nuclear domains, referred to as chromosome territories (CTs). The molecular mechanisms that underlie the formation and maintenance of CTs during the cell cycle remain largely unknown. Here, we have developed high-resolution chromosome paints to investigate CT organization in *Drosophila* cycling cells. We show that large-scale chromosome folding patterns and levels of chromosome intermixing are remarkably stable across various cell types. Our data also suggest that the nucleus scales to accommodate fluctuations in chromosome size throughout the cell cycle, which limits the degree of intermixing between neighboring CTs. Finally, we show that the cohesin and condensin complexes are required for different scales of chromosome folding, with condensin II being especially important for the size, shape, and level of intermixing between CTs in interphase. These findings suggest that large-scale chromosome folding driven by condensin II influences the extent to which chromosomes interact, which may have direct consequences for cell-type specific genome stability.

## Author summary

Eukaryotic genomes encode genetic information in their linear sequence, but appropriate expression of their genes requires chromosomes to fold into complex and spatially distinct three-dimensional structures known as chromosome territories (CTs). Despite the remarkable conservation of this organizational feature, we have a very limited molecular understanding of how chromosomes are spatially partitioned and functionally packaged into CTs in the nucleus. Here, we describe an efficient and scalable method of high-resolution chromosome painting using Oligopaints. We have generated Oligopaints to the entire non-repetitive portion of the *Drosophila* genome, using their relatively small genome to more easily capture a complete picture of chromosome organization during interphase. We demonstrate that levels of inter-chromosomal associations are remarkably consistent in a variety of cell types and throughout the cell cycle. We have also isolated the condensin II complex as a factor that regulates the level of inter-chromosomal associations during interphase. These findings have implications for many cellular processes that maintain genome stability, such as gene regulation and DNA repair.

## Introduction

Metazoan genomes are arranged into a nested hierarchy of structural features, ranging from small chromatin loops to larger insulated neighborhoods or topologically associated domains (TADs) [1–9]. TADs are believed to direct and insulate gene regulatory networks [10–12], which can engage in long-range interactions with each other, ultimately packaging chromosomes into sub-nuclear compartments termed chromosome territories (CTs).

CTs are a widespread feature of nuclear organization across a variety of cell types and species, as revealed by both fluorescence *in situ* hybridization (FISH) and chromosome-conformation-capture (3C)–based studies [5, 6, 13–17]. Recently, several studies have implicated the ring-shaped SMC (structural maintenance of chromosomes) complexes–cohesin and condensin–in the regulation of large-scale chromatin folding and CT formation [12, 18–20]. However, the contribution of each complex to local topology, large-scale chromatin folding, and chromosome individualization at single-cell resolution has been hindered by technical limitations. The consequence of CT loss during interphase also remains unclear. This is due, in part, to both the paucity of factors known to directly influence this level of organization and the difficulty in visualizing their effects at single cell resolution. However, CT intermixing has been theorized to influence the location and frequency of translocations [21–29] and the position of a gene within and between CTs seems to influence its access to the machinery responsible for specific nuclear functions, such as transcription, splicing, and DNA repair [21, 28].

Here, we leveraged the flexible, scalable Oligopaint FISH technology [30–32] to generate high-resolution chromosome paints to the entire *Drosophila* genome. Combined with a custom 3D segmentation pipeline, we provide a comprehensive picture of chromosome size, shape, and position at single-cell resolution. Our results show that various cell types in *Drosophila* harbor spatially partitioned CTs. Interestingly, widespread somatic homolog pairing in *Drosophila* results in homologs sharing a single CT, suggesting that homologous and heterologous chromosomes are distinguished at the cellular level in this species. Further, we characterize the differential roles of cohesin and condensin complexes in local chromatin compaction, large-scale chromatin folding, and CT formation. We find that cohesin and condensin II drive different scales of chromatin folding during interphase, with condensin II being especially important for large-scale interactions and the spatial partitioning of chromosomes. These findings indicate that condensin II-driven large-scale chromatin conformations during interphase influence the extent to which chromosomes interact, which has the potential to affect gene regulation and genome stability.

## Results

### Drosophila cells form robust CTs

**Fig 1:**
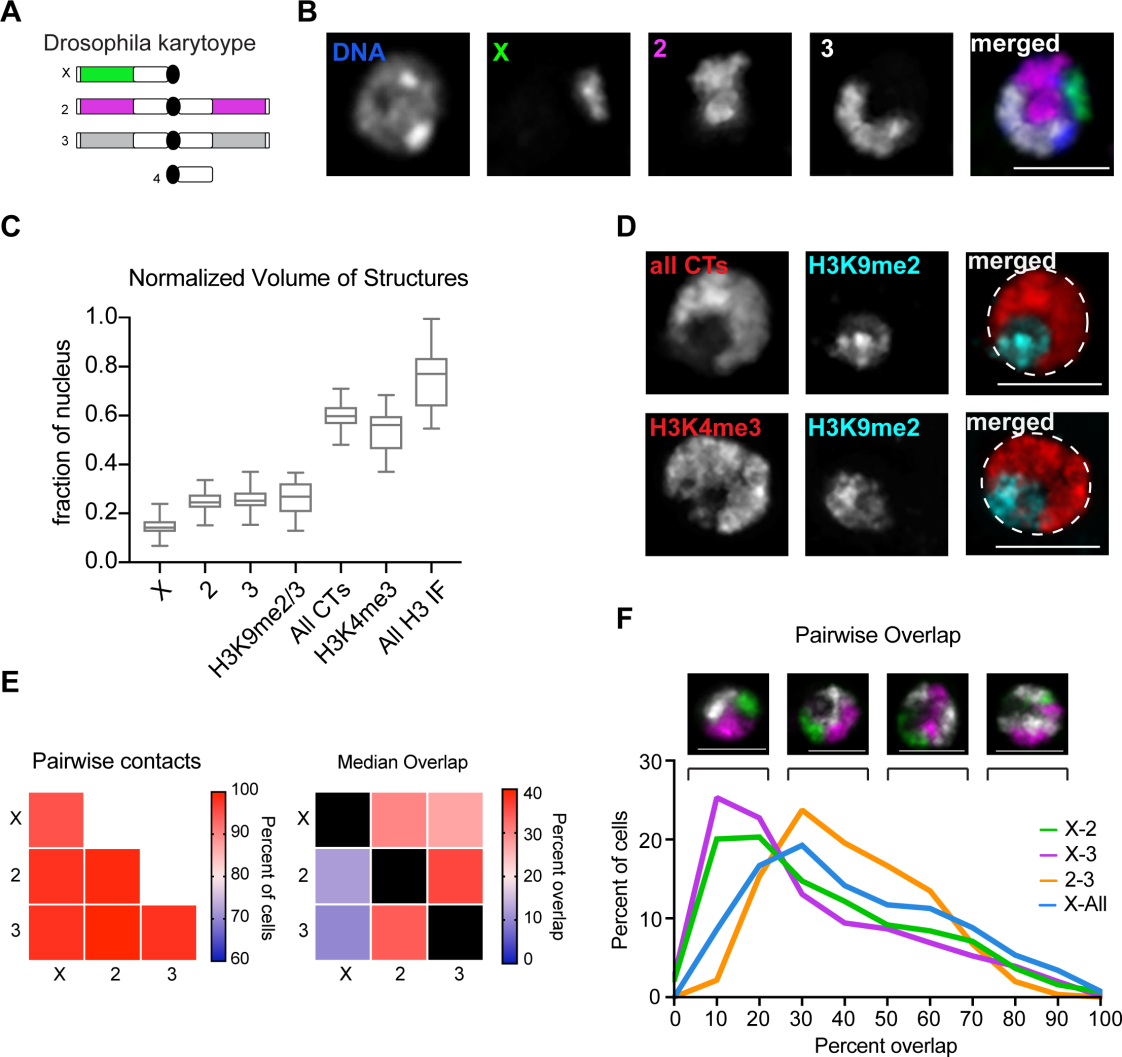
*Drosophila* Kc167 cells form robust CTs. (A) Schematic representation of *D*. *melanogaster* karyotype. The unlabeled heterochromatin is depicted in white, while euchromatin is depicted in green (X-chromosome), pink (chromosome 2), and gray (chromosome 3). (B) Representative Kc167 cell nucleus with Oligopaints labeling chromosomes X (green), 2 (pink), and 3 (gray). Total DNA (Hoechst stain) is shown in blue. Scale bar equals 5 μm. (C) Tukey box plot showing structure volume as a fraction of nuclear volume. X-axis denotes the structure being measured. (D) Top: Representative nucleus of IF/FISH in Kc167 cells with Oligopaints labeling chromosomes X, 2, and 3 all in red (all CTs), and anti-H3K9me2 IF in cyan. Bottom: IF to euchromatin (H3K4me3) in red and heterochromatin (H3K9me2) in cyan. Scale bar equals 5 μm. (E) Left: Heatmap of pairwise contact frequencies for a population of n>500 Kc167 cells. The diagonal boxes are whole-chromosome pairing frequencies. Right: Heatmap of median CT overlap for a population of n>500 Kc167 cells. Overlap is shown as a percentage of CT volume. Values in the bottom half of the heatmap are normalized to the structures listed along the bottom, while the top half are normalized to the structures listed on the left. (F) Top: Representative images of Kc167 cells with whole chromosome Oligopaints, showing the varying degrees of CT intermixing found in the population. Scale bar equals 5 μm. Bottom: histogram showing CT overlap as a percent of CT volume. Overlap between chromosomes X and 2, and X and 3, are shown as a percent of chromosome X CT volume. Overlap between chromosomes 2 and 3 is shown as a percent of chromosome 2 CT volume.

To determine whether immortalized *Drosophila* cell lines support CT formation, we selected the commonly used Kc167 cell line derived from late-stage embryos. Using Oligopaint FISH technology [30], we designed highly specific chromosome paints targeting the three major chromosomes in the *Drosophila* genome (X, 2, and 3). A total of 168,032 unique oligos were generated to label the non-repetitive portion of the genome, covering approximately 118 Mb. As shown in Fig 1, three-color Oligopaints targeting chromosomes X, 2, and 3 revealed that Kc167 cells form robust and spatially distinct CTs.

Using a computational image analysis pipeline to segment and measure the 3D volume of each chromosome signal [33], we found that each chromosome occupies a small fraction of the nucleus (median = 14–25%; Fig 1C). Notably, we did not find any cells where a single chromosome occupied more than 37% of the nucleus, indicating that chromosomes are stably compacted in Kc167 cells. Collectively, the total volume of FISH signal for chromosomes X, 2, and 3 (excluding overlap) ranged from 42–71% of the nucleus, similar to the fraction occupied by the euchromatic marker H3K4me3 (37–68%; Fig 1C and 1D). This result suggests that the entire non-repetitive portion of the genome can be efficiently labeled by Oligopaints. Additionally, each chromosome paint presented as a single FISH signal in >90% of nuclei (Fig 1B–1E), consistent with previously reported levels of euchromatic pairing in this cell type [34]. This finding demonstrates that homologous chromosomes share an individual territory in nearly all cells.

To determine the extent of intermixing between different CTs, we calculated the absolute pairwise contact frequencies between CTs, and the median inter-CT overlap volume based on voxel colocalization, presented as a fraction of CT volume. The overlap between each CT pair consistently peaked at 10–30% of the CT volume (Fig 1F). However, all cells exhibited some contact between chromosomes and we determined that all three chromosomes are in contact in >95% of cells (Fig 1E). This observation indicates that *Drosophila* Kc167 cells lack an inter-chromatin space that separates CTs, consistent with CT analysis of human lymphocytes [21].

To calculate how much each CT intermixes with the rest of the genome, we measured the volume of chromosome X that colocalized with all other chromosomes. We observed a large range of intermixing levels across the cell population with <5% of cells exhibited >90% overlap. However, on average, only 40% of the X chromosome volume was intermixed with the rest of the genome (Fig 1F). In summary, *Drosophila* Kc167 cells form robust CTs despite widespread somatic homolog pairing. These data also demonstrate that the whole-chromosome Oligopaints and 3D segmentation pipeline can detect and quantitate levels of chromosome compaction and intermixing in these cells.

### Chromosome arms form independent territories

**Fig 2:**
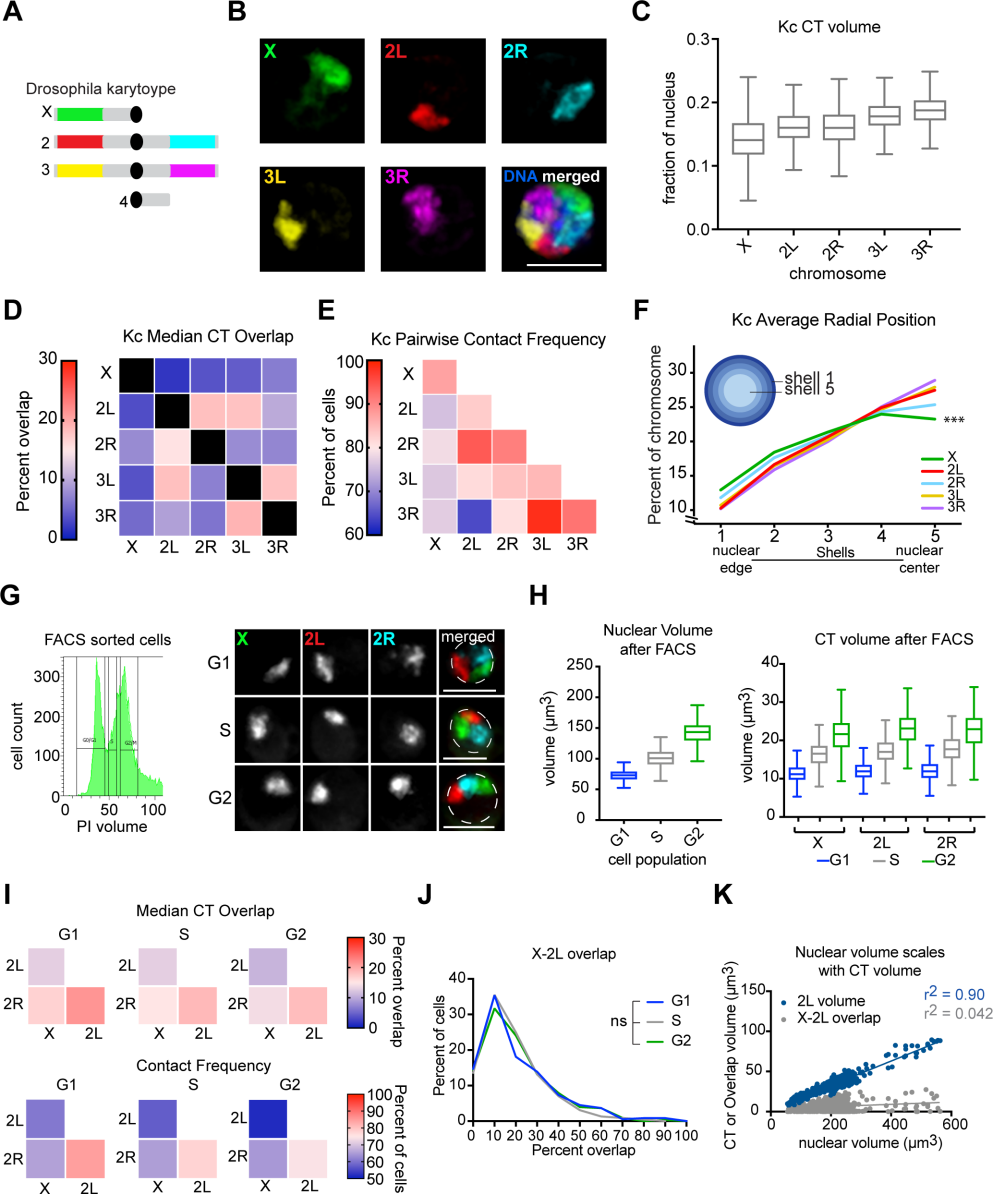
Chromosome arms form independent territories. (A) Schematic of chromosome arm-specific Oligopaints. (B) Representative Kc167 cell nucleus with Oligopaints labeling chromosomes X (green), 2L (red), 2R (cyan), 3L (yellow), and 3R (magenta). Total DNA (Hoechst stain) is shown in blue. Scale bar equals 5 μm. (C) Tukey box plot showing CT volumes as a fraction of nuclear volume. n>500 cells. (D) Heatmap showing median CT overlap for a population of n>500 Kc167 cells, where overlap is shown as a percentage of CT volume. Values in the bottom half of the heatmap are normalized to the structures listed along the bottom, while the top half are normalized to the structures listed on the left. (E) Heatmap showing pairwise contact frequencies for a population of n>500 Kc167 cells. The diagonal boxes represent whole-chromosome pairing frequencies. (F) Radial position of chromosomes in the nucleus determined by shell analysis with five shells of equal volume, where shell 1 is the closest to the nuclear periphery and shell 5 is the nuclear center. n>500 cells. ***p<0.0001, Mann-Whitney test. (G) Left: plot of DNA content in Kc167 cells after FACS. Right: Representative nuclei after FACS with Oligopaints labeling chromosomes X (green), 2L (red), and 2R (cyan). Dashed lines represent nuclear edge. Scale bar equals 5 μm. (H) Left: Tukey box plot showing nuclear volumes of G1, S, and G2 phased cells after FACS sorting, determined by Hoechst stain. Right: Tukey box plot showing CT volumes after FACS sorting. The data shown represent one technical replicate (n = 220–350 cells per cell cycle phase). These data were confirmed by two additional technical replicates. (I) Top: heatmaps showing median CT overlap for a population of n>200 FACS sorted Kc167 cells, where overlap is shown as a percentage of the structures listed along the bottom. Bottom: heatmaps showing pairwise contact frequencies for a population of n>200 FACS sorted Kc167 cells. (J) Histogram showing X-2L CT overlap as a percent of X CT volume after FACS. Binned data from a single technical replicate are shown (n>200 cells). These results were confirmed by at two additional technical replicates. (K) Scatter plot of nuclear volume (X-axis) versus 2L CT volume or X-2L overlap volume (Y-axis). Chromosome 2L volume data are shown in blue, while X-2L overlap data are shown in gray. n = 835 cells.

Whole-chromosome painting of chromosome 2 and 3 each often revealed two spatially distinct substructures. Based on this observation, we hypothesized that the chromosome arms form their own territories during interphase. We segmented our Oligopaint libraries to distinctly label the five major chromosome arms in *Drosophila* (Fig 2A and 2B). The FISH signal volumes were similar for each arm, consistent with their comparable genomic size [35], and each 3D chromosomal arm signal occupied on average 13–18% of the nucleus (Fig 2C). The two arms of the metacentric chromosomes, chromosomes 2 and 3 (2L-2R and 3L-3R, respectively), had median overlap fractions at or below 20% of their volume, indicating that they were spatially separated from each other and that the chromosome arms form their own CTs (Fig 2D). These findings are consistent with Hi-C results in *Drosophila* and mammalian cells [6, 36], suggesting that centromeric regions reduce associations between DNA located on opposite chromosomal arms.

The median overlap fraction of one chromosome arm with any other chromosome arm was between 5–20% of the CT volume (Fig 2D), similar to that observed for whole chromosomes (Fig 1E and 1F). In contrast to whole chromosomes, however, not all arm CTs are in contact in every cell (Fig 2E). While we observed slight differences in contact and overlap between the various CT pairs, these differences were small and mostly negligible, suggesting that CTs are not preferentially arranged relative to each other in Kc167 cell nuclei. The notable exception to this was the increased overlap detected between chromosomes 2L and 3L compared to other CT pairs. We used our arm-specific Oligopaints to karyotype mitotic chromosome spreads and found that the preferential association between 2L and 3L correlates with a stable chromosome translocation in Kc167 cells (S1 Fig). This observation indicates that translocated regions join the CT of their inserted chromosome rather than their original chromosome arm. Further, this result demonstrates that Oligopaints have sufficient sensitivity to detect translocation events in interphase through 3D overlap.

Next, we assayed CT formation *in vivo* in diploid hemocytes and in five additional immortalized cell lines that differed in sex (X versus XY), morphology, tissue of origin (i.e., embryonic, hemocyte, larval imaginal disc, larval central nervous system), and karyotype/ploidy (i.e., diploid, polyploid/aneuploid). In all cases, we observed similar extents of CT intermixing to that observed in Kc167 cells (S2 Fig). Karyotype analysis of these cell lines revealed that all CT pairs exhibiting higher levels of overlap have stable or frequent chromosome translocations between the overlapping chromosome arms (S1 Fig). Importantly, there were no preferential chromosomal arm interactions observed in BG3 cells, which have a normal diploid karyotype (S1 Fig). These results support the hypothesis that *Drosophila* chromosomes do not have preferential neighbors.

To determine if CTs have preferential radial positions in the nucleus, we performed a 3D shell analysis and quantified the percentage of chromosome signals in each concentric shell (Fig 2F). All chromosome arms were found to span all shells, suggesting all chromosomes have regions that are peripheral and central in the nucleus [37]. However, the majority of chromosome signals in all cell lines were located in the center shell (shell 5, Fig 2F and S3 Fig). This is consistent with our labeling of the single-copy and euchromatic-enriched portion of chromosomes, which has a tendency to localize toward the nuclear interior [38]. Consistent with this interpretation, chromosome X, which has a lower gene density compared with autosomes [35], was the most peripheral in the majority (5/7) of cell lines tested (S3 Fig). Similar results have been observed for gene-poor chromosomes in mammalian nuclei [39]. Collectively, these data demonstrate that *Drosophila* cells form robust, arm-specific CTs in a variety of cell types.

### CTs remain stable throughout the cell cycle

To investigate the impact of the cell cycle on CT size and intermixing, Kc167 cells were sorted by FACS into G_1_-, S-, and G_2_-phase groups based on their DNA content (Fig 2G). Consistent with the replication-associated doubling of genomic content, chromosome painting revealed increased nuclear and chromosome volumes as cells progressed through the cell cycle (Fig 2G and 2H). Despite this increase in volume, the distribution and median level of intermixing were nearly uniform across all three groups, and they did not differ significantly from unsorted cells (Fig 2I and 2J).

Additionally, we observed a weak correlation between the volume of chromosome 2L and its level of intermixing with chromosome X in unsorted cells (R^2^ = 0.04; Fig 2K). Instead, the chromosome volume was highly correlated with its corresponding nuclear volume (R^2^ = 0.90) (Fig 2K). These results support the hypothesis that the extent of chromosome intermixing is regulated independently of CT size. Further, these data suggest that the nucleus can scale to accommodate fluctuations in CT size throughout the cell cycle, which limits the degree of intermixing between each CT pair.

### Condensin II regulates the levels of inter-chromosomal associations

**Fig 3:**
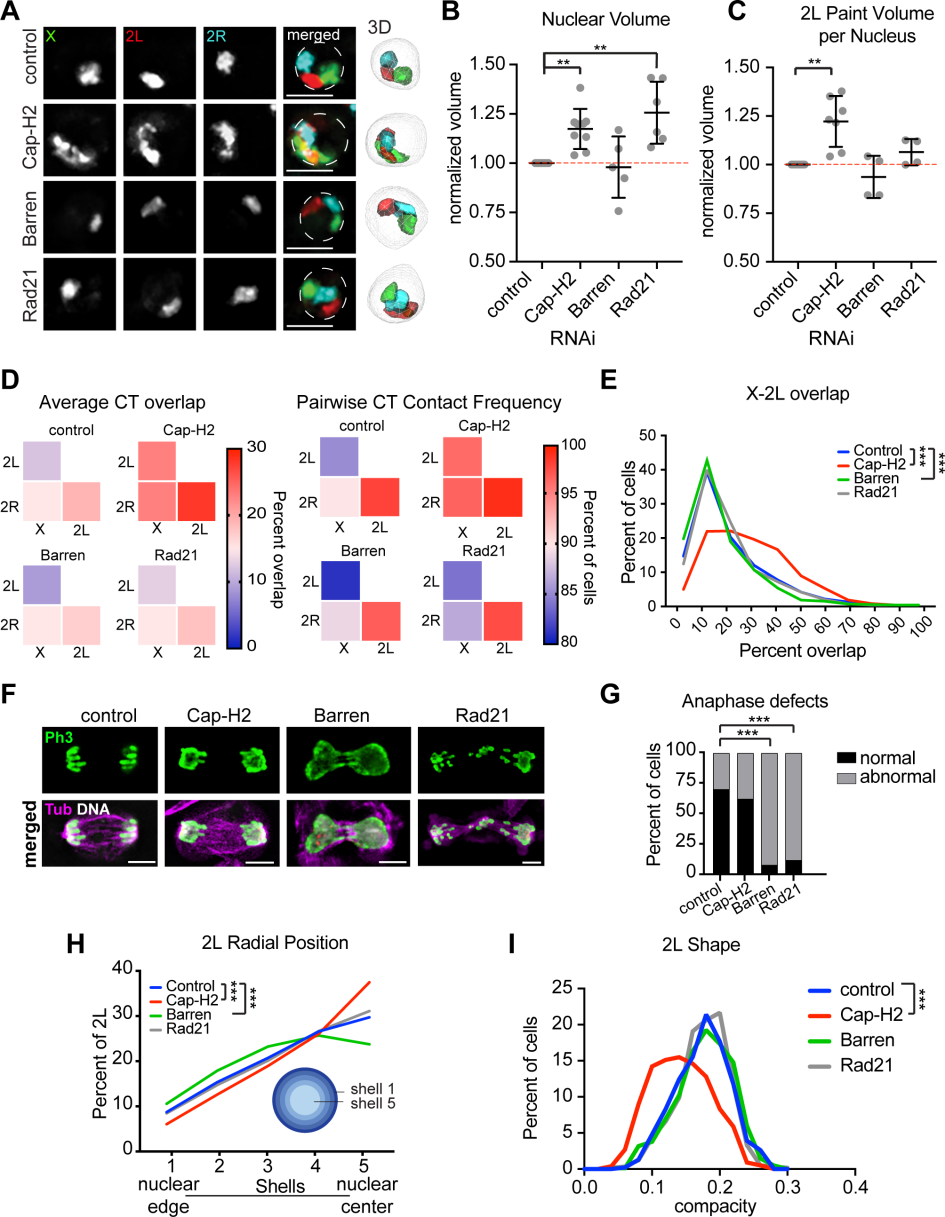
Condensin II regulates levels of inter-chromosomal associations. (A) Left: Oligopaints labeling chromosomes X (green), 2L (red), and 2R (cyan) on representative Kc167 cell nuclei depleted of Brown (control), Condensin II (Cap-H2), Condensin I (Barren), or Cohesin (Rad21). Dashed lines represent nuclear edge. Scale bar equals 5 μm. n>500 cells per RNAi. Right: 3D renderings of segmented structures. (B) Dot plot showing nuclear volume after RNAi normalized to control cells. Each dot represents the average of a biological replicate with n>500 cells. **p = 0.005; t-test. (C) Dot plot showing total 2L Oligopaint volume per nucleus normalized to control cells. Each dot represents the average of a biological replicate with n>500 cells. **p = 0.005; t-test. (D) Left: heatmaps showing average CT overlap for >3 biological replicates, each with n>500 cells. Overlap is shown as a percentage of the structures listed along the bottom. Right: heatmaps showing absolute pairwise contact frequencies for >3 biological replicates, each with n>500 cells. (E) Histogram showing X-2L CT overlap as a percent of X CT volume. Binned data from a single biological experiment are shown (n>500 cells). These results were confirmed by at least two additional biological replicates. ***p < 0.0001; Mann-Whitney test. (F) Representative IF images showing anaphase cells from the knockdowns in (A-I). Anti-PH3S10 (mitotic marker) antibody is shown in green, and anti-tubulin in magenta. DNA (Hoechst stain) is shown in white. (G) Quantification of anaphase defects shown in (F). Data shown are from a single biological experiment (n>50 anaphase cells). These results were confirmed by two additional biological replicates. ***p < 0.0001; Fisher’s Exact Test. (H) Average 2L radial position in the nucleus determined by shell analysis with five shells of equal volume, where shell 1 is the closest to the nuclear periphery and shell 5 is the nuclear center. Averages from a single biological experiment are shown (n>500 cells). These results were confirmed by at least two additional biological replicates. ***p < 0.0001; Mann-Whitney test. (I) Histogram showing the binned distribution of 2L shape from a single biological experiment (n>500). Higher compacity values indicate a more spherical structure. These results were confirmed by at least two additional biological replicates. ***p < 0.0001; Mann-Whitney test.

Recently, SMC protein complexes have been implicated in the formation of CTs in yeast, tetrahymena, and post-mitotic polytene nuclei in *Drosophila* [19, 20, 40]. Therefore, we tested the individual contribution of the three major SMC complexes (cohesin, condensin I, and condensin II) to chromosome size and intermixing in Kc167 cells using our arm-specific Oligopaints. First, we depleted the kleisin subunits specific to cohesin (Rad21), condensin I (Barren), or condensin II (Cap-H2) using RNAi, and then labeled chromosomes X, 2L, and 2R with Oligopaints (Fig 3A and S4 Fig). The average nuclear volumes increased significantly (p ≤ 0.005) following either Rad21 or Cap-H2 knockdown, which is consistent with chromosome folding defects (Fig 3B). However, only Cap-H2 depleted cells exhibited significantly increased chromosome volumes (p ≤ 0.005; Fig 3C).

We next analyzed the overlap volume between different CT pairs. Neither Rad21 (cohesin-specific) nor Barren (condensin I–specific) depletion affected the average intermixing volume for any of the tested CT pairs, despite retaining only 15% and 40% of transcript levels, respectively (Fig 3D and 3E and S4A Fig). Defects in heterochromatin clustering and high levels of chromosome missegregation during mitosis confirmed the loss of SMC complex function due to Rad21 and Barren depletion (Fig 3F and 3G and S3B and S3C Fig). Together, these findings suggest that levels of inter-chromosomal associations in *Drosophila* are not significantly affected by depletion of cohesin or condensin I, and are not affected by mitotic defects or aneuploidy, further highlighting the tenacity of CT formation in cycling cells.

By contrast, Cap-H2 (condensin II) depletion dramatically increased the levels of contact and intermixing between every tested CT pair (Fig 3D and 3E). Pairwise contact of all CTs was observed in >96% of Cap-H2-depleted cells (Fig 3D). The overlap between chromosomes X and 2L shifted from a sharp peak at 12% of the volume of chromosome X in control cells to a broad spread between 20% and 50% (Fig 3E) and the percentage of cells with >30% overlap increased greater than 2-fold. We estimate the total intermixing of the genome following condensin II depletion to be 80–100%. Similar results were observed following the depletion of Cap-D3 and SMC2, other condensin II subunits, in Kc167 cells (S4F–S4K Fig) and following Cap-H2 depletion in BG3 cells (S5 Fig). These results suggest that the entire condensin II holocomplex is essential for CT formation in multiple cell types.

The radial position and shape of chromosomes were also altered following Cap-H2 depletion (Fig 3H and 3I), further highlighting the level of disorganization in these nuclei. Importantly, Cap-H2 depletion did not lead to increased chromosome missegregation during mitosis, and did not significantly increase the frequency of chromosome rearrangements based on five-color arm-specific karyotyping of mitotic spreads (Fig 3F and 3G and S4D Fig). Therefore, our findings suggest that increased chromosome intermixing following the loss of condensin II occurs specifically during interphase and is not a result of genome instability.

### Condensin II drives compaction and separation of whole-chromosomes during interphase

**Fig 4:**
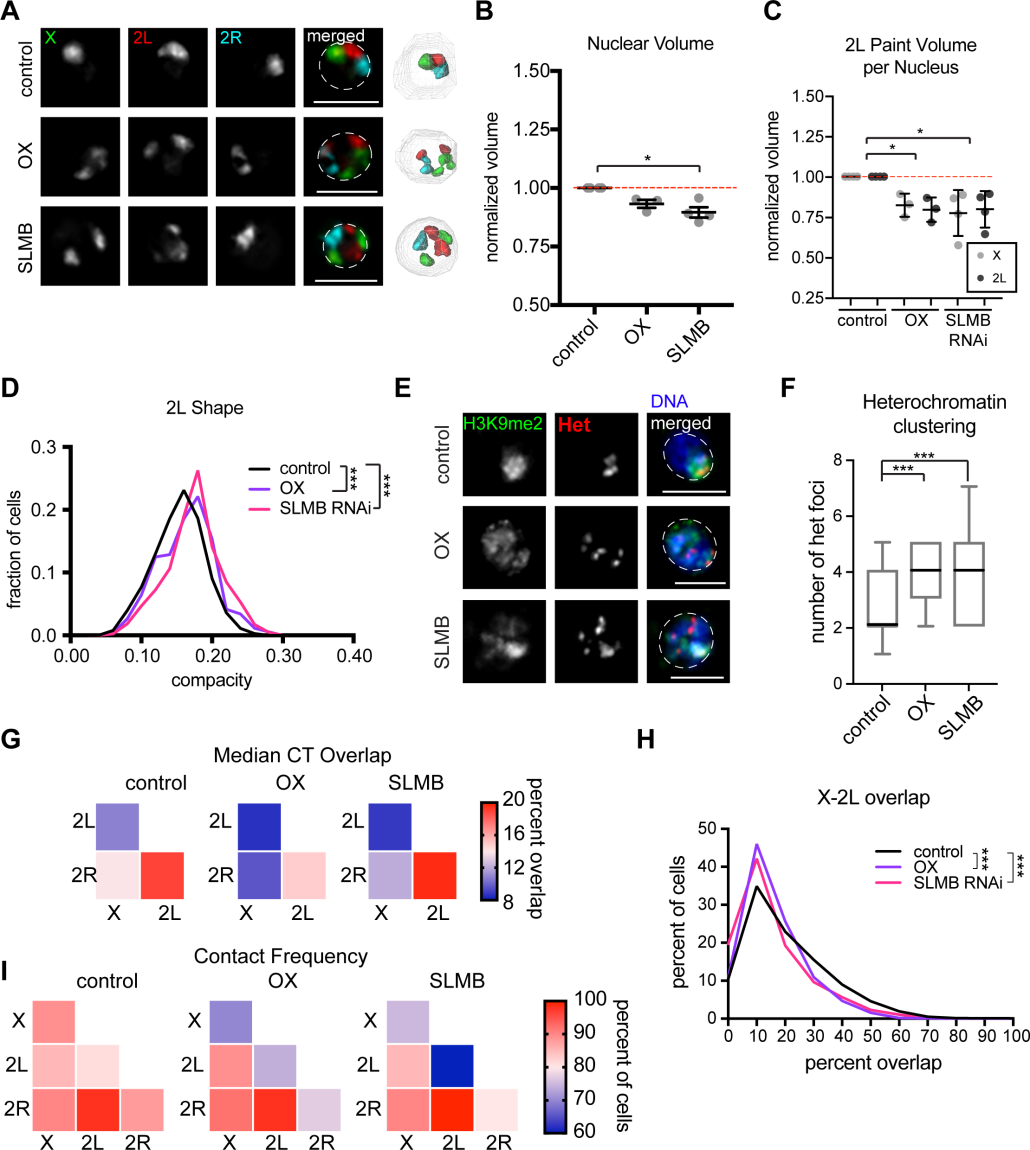
Condensin II is sufficient to drive whole-chromosome separation. (A) Left: Oligopaints labeling chromosomes X (green), 2L (red), and 2R (cyan) on representative Kc167 cell nuclei depleted of Brown (control) or the condensin II regulator SLMB, or stably expressing a copper sulfate-inducible Cap-H2-GFP construct (OX). Dashed lines represent the nuclear edge. Scale bar equals 5 μm. n>500 cells per condition. Right: 3D renderings of segmented structures. (B) Dot plot showing nuclear volume normalized to control. Each dot represents the average of a biological (SLMB RNAi) or technical (Cap-H2 OX) replicate with n>500 cells. *p = 0.02; t-test. (C) Dot plot showing 2L Oligopaint volume normalized to control. Each dot represents the average of a biological (SLMB RNAi) or technical (Cap-H2 OX) replicate with n>500 cells. *p = 0.04; t-test. (D) Histogram showing the binned distribution of 2L shape from a single biological (SLMB RNAi) or technical (Cap-H2 OX) replicate with n>300 cells. Higher compacity values indicate a more spherical structure. These results were confirmed by at least two additional biological or technical replicates. ***p < 0.0001; Mann-Whitney test. (E) IF/FISH on representative Kc167 cell nuclei depleted of Brown (control) or SLMB, or stably expressing a copper sulfate-inducible Cap-H2-GFP construct (OX). Heterochromatin is labeled with anti-H3K9me2 antibody (green) and heterochromatin FISH probes (Het) labeling AATAT, AATAG, AACAC, 359, and dodeca in red. Dashed lines represent the nuclear edge. DNA (Hoechst stain) is shown in blue. Scale bar equals 5 μm. n>300 cells per condition. (F) Tukey box plot of the number of Het foci shown in (E), showing the mean (black line) and distribution (minus outliers). Data shown are from a single biological (SLMB RNAi) or technical (Cap-H2 OX) replicate (n>300 cells each). These results were confirmed by two additional biological or technical replicates, respectively. ***p < 0.0001; Mann-Whitney test. (G) Heatmap showing median CT overlap for a population of n>500 cells per condition, where overlap is shown as a percentage of the structures listed along the bottom. (H) Histogram showing X-2L CT overlap as a percent of X CT volume. Binned data from a single biological or technical replicate are shown (n>500 cells). These results were confirmed by at least two additional biological or technical replicates. ***p < 0.0001; Mann-Whitney test. (I) Heatmap showing pairwise contact frequencies for a population of n>500 cells per condition. The diagonal boxes are whole-chromosome pairing frequencies.

To assess the ability of condensin II to drive CT formation, we ectopically overexpressed the Cap-H2 subunit in Kc167 cells using an inducible promoter, and labeled chromosomes X, 2L, and 2R with Oligopaints (Fig 4A and S6A Fig). Previous reports have indicated that Cap-H2 acts as a limiting subunit of the condensin II complex and is sufficient to drive its activity during interphase [19, 41, 42]. At 24-hours post-induction, nuclear and chromosome volumes decreased and chromosome shapes became more spherical compared to controls, indicating hyper-compacted chromosomes (Fig 4B–4D). Similar results were observed following depletion of the condensin II regulator SLMB (Fig 4, S5A, S5B, S6B and S6C Fig), which is part of an SCF ubiquitin ligase complex that targets Cap-H2 for degradation during interphase [41, 43]. Consistent with these reports, increased Cap-H2 levels following either SLMB depletion or Cap-H2 overexpression increased the number of FISH signals labeling pericentric heterochromatin, indicating heterochromatin dispersal [19, 41, 43](Fig 4E and 4F).

In addition, Cap-H2 overexpression and SLMB depletion both decreased the overlap between chromosomes X, 2L, and 2R (Fig 4G and 4H). Combined with heterochromatin dispersal, this suggests that whole chromosomes are more spatially separated when Cap-H2 levels are increased. The overlap between chromosomes 2L and 2R also decreased (Fig 4G), suggesting that condensin II strengthens the boundary between metacentric chromosome arms by decreasing cross-boundary interactions. Finally, whole-chromosome unpairing of homologs increased significantly following SLMB depletion and Cap-H2 overexpression (Fig 4I, p = 0.03), indicating that the homologs are forming their own CTs more frequently. Collectively, these results suggest that increased condensin II levels can increase interphase CT partitioning and chromosome compaction, producing smaller ‘super territories’ that are more spatially separated.

### Heterochromatin clustering is dispensable for CT formation

**Fig 5:**
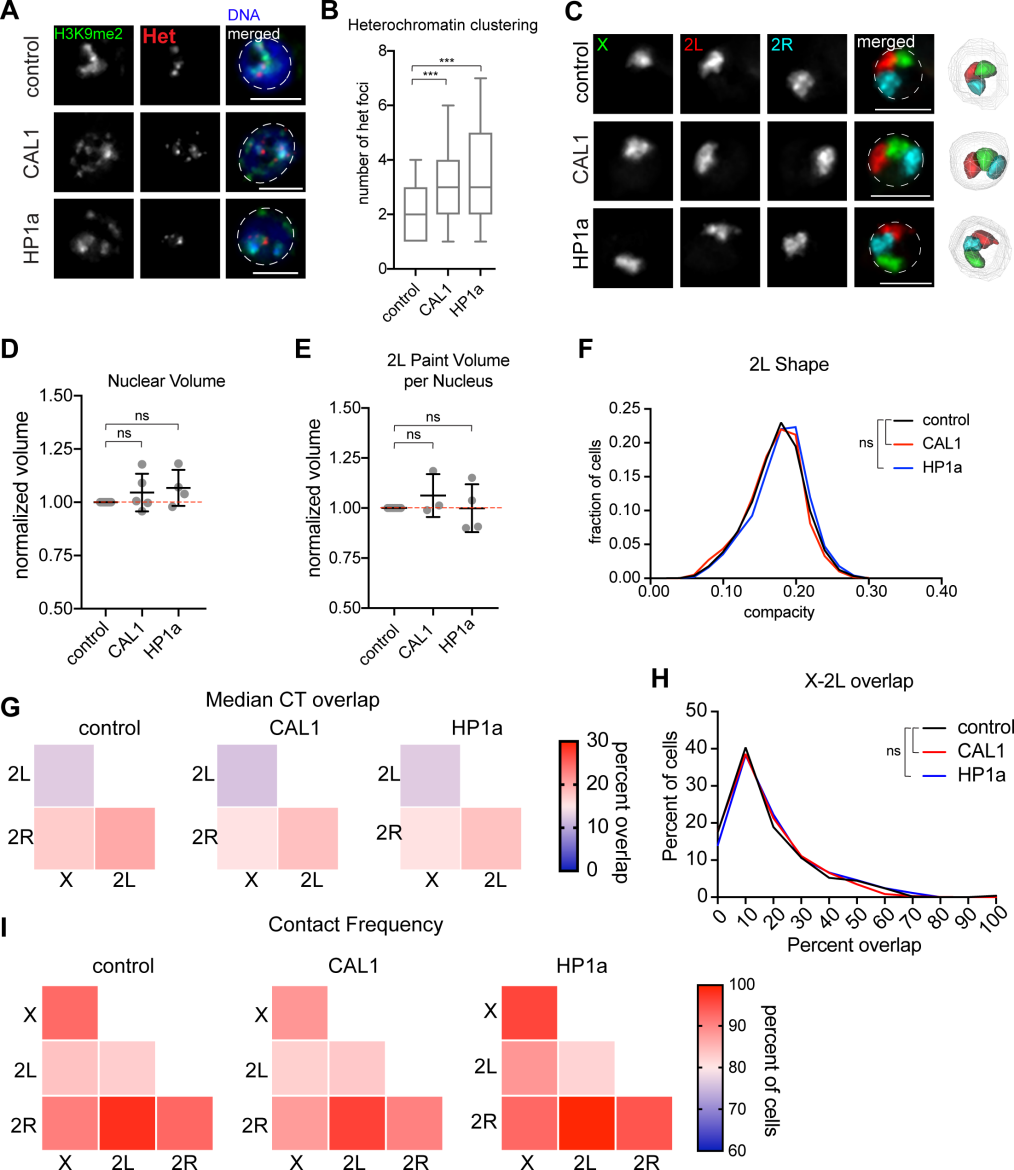
Heterochromatin clustering is dispensable for CT formation. (A) IF/FISH on representative Kc167 cell nuclei depleted of Brown (control), CAL1, or HP1a. Heterochromatin is labeled with anti-H3K9me2 antibody (green) and heterochromatin FISH probes (Het) labeling the AATAT, AATAG, AACAC, 359, and dodeca satellites in red. DNA (Hoechst stain) is shown in blue. Dashed lines represent the nuclear edge. Scale bar equals 5 μm. n>500 cells per condition. (B) Tukey box plot of the number of Het foci shown in (A). Data shown are from a single biological replicate (n>500 cells each). These results were confirmed by two additional biological replicates. ***p < 0.0001; Mann-Whitney test. (C) Left: Oligopaints labeling chromosomes X (green), 2L (red), and 2R (cyan) on representative Kc167 cell nuclei depleted of Brown (control), CAL1, or HP1a. Dashed lines represent the nuclear edge. Scale bar equals 5 μm. n>500 cells per condition. Right: 3D renderings of segmented structures. (D) Dot plot showing nuclear volume normalized to control. Each dot represents the average of a biological replicate with n>500 cells. The differences shown are not significant (t-test). (E) Dot plot showing 2L Oligopaint volume normalized to control. Each dot represents the average of a biological replicate with n>500 cells. The differences shown are not significant (t-test). (F) Histogram showing the binned distribution of 2L shape from a single biological replicate with n>500 cells. Higher compacity values indicate a more spherical structure. These results were confirmed by at least two additional biological replicates. The differences shown are not significant (Mann-Whitney test). (G) Heatmap showing median CT overlap for a population of n>500 cells per condition, where overlap is shown as a percentage of the structures listed along the bottom. (H) Histogram showing X-2L CT overlap as a percent of X CT volume. Binned data from a single biological replicate are shown (n>500 cells). These results were confirmed by at least two additional biological replicates. The differences shown are not significant (Mann-Whitney test). (I) Heatmap showing pairwise contact frequencies for a population of n>500 cells per condition. The diagonal boxes are whole-chromosome pairing frequencies.

Considering Cap-H2 overexpression leads to both whole-chromosome compaction and heterochromatin dispersal, we next determined if the disruption of heterochromatin organization indirectly alters the folding of whole chromosomes into CTs. We tested the contributions of the heterochromatic protein HP1a and the centromeric chaperone protein CAL1 to CT position, size, and overlap. Consistent with previous reports, we found that HP1a and CAL1 depletion each caused heterochromatin dispersal by both H3K9me2 staining and FISH targeting pericentric regions (Fig 5A and 5B) [43–45]. Efficient knockdown of each factor was further confirmed by RT-qPCR and IF (S6D–S6F Fig). Remarkably, Oligopaints targeting chromosomes X, 2L, and 2R revealed no significant change in nuclear or chromosome volume following depletion of either HP1a or CAL1 (Fig 5C–5E), suggesting heterochromatin dispersal does not necessarily change the level of compaction of whole chromosomes during interphase. Furthermore, knockdown of HP1a or CAL1 did not alter the shape, level of intermixing, contact frequency, or radial positioning of chromosomes in the nucleus (Fig 5F and 5I). These results indicate that the structural integrity and spatial organization of centromeres and heterochromatin are each dispensable for the positioning and formation of CTs. Importantly, the level of intermixing and contact frequency between chromosomes 2L and 2R were also unchanged relative to controls following CAL1 depletion, which abrogates CID/CENPA deposition (Fig 5G, 5I and S6E Fig). This suggests that these centromere proteins are not critical components of the boundary that abrogates interactions between arms of metacentric chromosomes.

### Condensin II and cohesin drive different scales of chromatin folding during interphase

Our finding that condensin II drives CT formation in cycling cells implicates chromatin compaction in intermixing regulation. However, we found no correlation between chromosome volume and the level of CT intermixing, even following Cap-H2 depletion (r^2^ = 0.052, S4E Fig) or overexpression (r^2^ = 0.085, S6C Fig), indicating that changes in local compaction may not fully describe condensin’s role in CT formation. Instead, we hypothesized that condensin II may play a role in higher-order chromosome folding. This notion is consistent with our observation of amorphous and significantly less spherical chromosome shapes following condensin II depletion (Fig 3A and 3I), and smaller, more spherical chromosome shapes following condensin II overexpression (Fig 4C and 4D).

**Fig 6:**
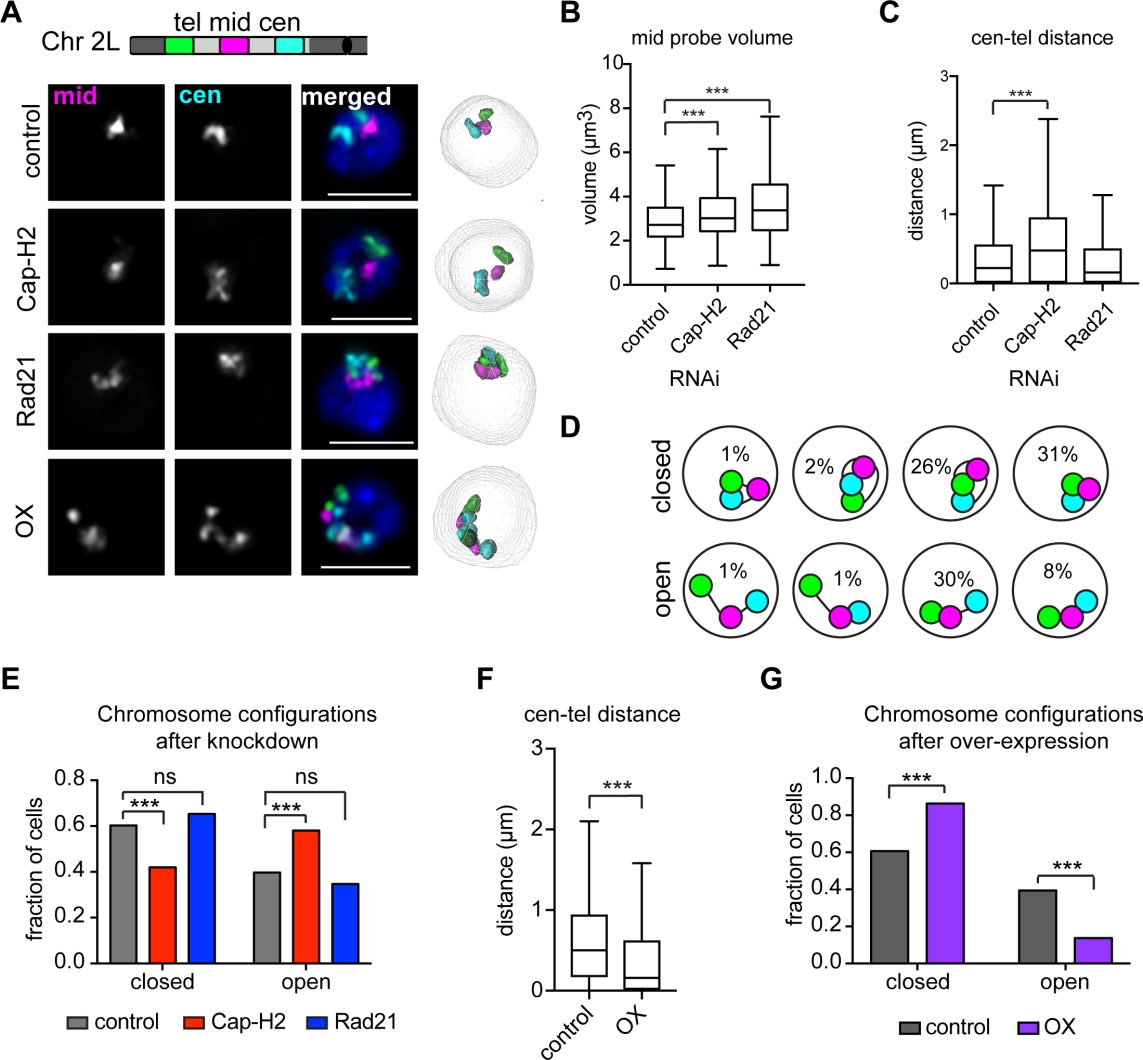
Condensin II and cohesin drive different scales of chromatin folding during interphase. (A) Top: Schematic of three-color Oligopaints. Three 3–4 Mb sized probes were tiled along chromosome 2L. The centromere-proximal probe (cen) is shown in cyan, the telomere-proximal probe (tel) is shown in green, and the middle probe (mid) is shown in pink. Bottom: Representative nuclei with three-color FISH are shown from control (Brown), Cap-H2, or Rad21 depleted cells, or cells over-expressing Cap-H2 (OX). DNA (Hoechst) is shown in blue. Scale bars equal 5 μm. 3D rendering of structures are shown on the right. (B) Tukey box plot showing the volume of the mid signal probe after RNAi. The data shown are from a single biological replicate (n>500 cells). These results were confirmed by at two additional biological replicates. ***p < 0.0001; Mann-Whitney test. (C) Tukey box plot showing the minimal edge-to-edge distance between the cen and tel probes after RNAi. The data shown are from a single biological replicate (n>500 cells). These results were confirmed by at two additional biological replicates. ***p < 0.0001; Mann-Whitney test. (D) Schematic representation of eight possible chromosome configurations detected using this approach. Configurations were classified as either ‘closed’ (cen and tel-proximal probes touching), or open (cen and tel-proximal probes not touching). The percentage of untreated Kc167 cells with a particular 2L configuration is noted inside the corresponding circle. (E) Bar graph showing the fraction of cells with chromosome 2L in either an open or closed configuration after RNAi. The data shown are from a single biological replicate (n>500 cells). These results were confirmed by at two additional biological replicates. ***p < 0.0001; Fisher’s exact test. (F) Tukey box plot showing the minimal edge-to-edge distance between the cen and tel probes before in control cells and after Cap-H2 overexpression (OX). The data shown are from a single biological replicate (n>500 cells). These results were confirmed by at two additional technical replicates. ***p < 0.0001; Mann-Whitney test. (G) Bar graph showing the fraction of cells with chromosome 2L in either an open or closed configuration in control cells and after Cap-H2 overexpression (OX). The data shown are from a single technical replicate (n>500 cells). These results were confirmed by at two additional technical replicates. ***p < 0.0001; Fisher’s exact test.

To simultaneously assess both local and long-range chromosome folding, we developed three-color Oligopaints that created a banding pattern across chromosome 2L. This labeling scheme allowed us to trace the path of interphase chromosomes. The three Oligopaint probes targeted 3–4 Mb domains located near the centromere, telomere, and chromosome arm center, while leaving 4 Mb of unlabeled DNA between each domain (Fig 6A). Following Cap-H2 depletion, the volume of each signal and the 3D distance between each pairwise signal combination increased compared to controls, consistent with a loss of intra-chromosomal interactions and subsequent chromatin decompaction (Fig 6A–6C). Importantly, signal volumes were also increased following Rad21 depletion (Fig 6A and 6B), supporting the role of cohesin in local chromatin topology [12, 46]. These results, combined with the finding that Rad21 depletion does not lead to CT loss, support the hypothesis that changes in local chromatin topology are not sufficient to disrupt CT formation.

Next, we analyzed large-scale chromosome folding by measuring the contact patterns between the three-color Oligopaint probes. While there are eight possible chromosome configurations with this painting scheme, we found that 87% of chromosomes adopt one of three configurations in control cells (Fig 6D and S7A Fig). In particular, the majority of control cells (60%) showed contact between the distal and proximal regions of chromosome 2L (Fig 6D). We classified this subgroup as “closed” configurations. “Open” chromosome configurations were defined as lacking this long-range interaction. Interestingly, the same frequency and pattern of chromosome configurations were observed in Kc167 cells after FACS into G1- and G2-subpopulations, and in BG3 cells (S7B and S7C Fig). Therefore, we conclude that these large-scale configurations represent stable folding schemes for *Drosophila* chromosome 2L.

Cap-H2 depletion significantly reduced interactions between the proximal and distal domains, increasing the frequency of open versus closed configurations across the cell population (Fig 6E). Additionally, Cap-H2 overexpression reduced the 3D distance between signals and increased the frequency of closed configurations compared to controls (Fig 6F and 6G), indicating that condensin II activity is necessary and sufficient for large-scale intra-chromosomal interactions that promote proximity of the distal and proximal regions of the chromosome. Conversely, cells depleted of Rad21 showed no changes in the contact frequency of distal and proximal domains despite exhibiting local chromatin decompaction (Fig 6A–6E). Further, Rad21-depleted cells maintained the wild-type pattern of chromosome folding configurations (S7D Fig). These data indicate that while both condensin II and cohesin are important for local chromosome topology, condensin II activity defines the long-range folding scheme of interphase chromosomes.

## Discussion

In this study, we demonstrate that *Drosophila* cells harbor spatially distinct CTs and found remarkably consistent levels of intermixing in a variety of cell types and throughout the cell cycle. While the vast majority of cells showed contact between all three major chromosomes, we were able to measure that, on average, only 40% of the *Drosophila* genome is intermixed (not accounting for homologous chromosomes). This is strikingly similar to the estimate of 40–46% CT intermixing in human lymphocytes [21], possibly indicating a widespread and conserved restraint on inter-chromosomal interactions. However, we note that a small population of cells do exhibit >90% overlap between neighboring CTs. The fate of these cells will be important to explore in the future.

Further, we identified the condensin II complex as an essential factor for CT formation in cycling cells. These results are consistent with those reported on condensin in yeast, tetrahymena, and post-mitotic polytene cells of *Drosophila* [19, 20, 40]. These data are also in line with previous work showing that condensin II serves as an ‘anti-pairing’ factor that disrupts pairing interactions and separates homologous loci [42, 43, 47]. Additionally, we showed that condensin II overexpression can further compact chromosomes and reduce the level of CT intermixing. Together, these data highlight the highly conserved role of the condensin II complex in controlling the level of inter-chromosomal associations in eukaryotic cells.

If condensin II has the capacity to spatially separate homologous and heterologous chromosomes, how does somatic pairing persist in *Drosophila* cells that have CTs? One possibility is that pairing interactions are established prior to CT formation and thus, homologous chromosomes would be folded in concert. This would be consistent with some persistence of homolog pairing through mitosis [34, 48] and suggests a model in which chromosomes are folded into CTs through post-mitotic condensin II activity. In addition, pairing interactions may require additional condensin activity to separate homologous versus heterologous interactions. Indeed, our studies showed that condensin II overexpression increases whole-chromosome unpairing in Kc167 and BG3 cells. We speculate that interphase condensin II levels and thus inter-chromosomal associations are tightly regulated, and could be modified in a cell-type-specific manner. For instance, in contrast to virtually all other cell types in *Drosophila*, homologous chromosomes in germline stem cells remain unpaired throughout development [49]. This separation between homologs could potentially reflect increased levels of condensin II activity and may indicate that inter-chromosomal associations are reduced to protect the stem-cell population from potentially deleterious rearrangements. Indeed, previous work has shown that different extents of chromosome intermixing correlate with translocation frequencies–both those occurring naturally in the human population and those induced experimentally in human and mouse lymphocytes [21, 50, 51]. Therefore, an alteration in condensin II activity and subsequent CT intermixing levels has the potential to influence the location and frequency with which translocations occur. Intriguingly, mice carrying a hypomorphic allele of *cap-H2* were recently shown to frequently develop T-cell lymphomas with highly rearranged chromosomes in the transformed cells [52]. It will be important to determine whether this increased genome instability is associated with increased CT contact prior to the rearrangement event.

When accounting for the popular model of loop extrusion [53] and the stabilizing function of SMC complexes [54–58], condensin II activity could potentially fold whole chromosomes into a configuration that limit their interactions with the rest of the genome. While the nature of these interactions remains unknown, they are clearly distinct from cohesin-driven interactions given that cohesin depletion does not significantly change intermixing levels in *Drosophila* or yeast [59]. Consistent with this hypothesis, a recent study demonstrated that depletion of the cohesin complex in mammals eliminates chromatin looping and TAD formation but does not disrupt long-range interactions between similar chromatin states, highlighting the notion that local insulation and higher-order folding must rely on distinct molecular determinants [12]. Combined with our findings that large-scale configurations are stable throughout the cell cycle and require condensin II activity, we propose that condensin II drives long-range interactions that are established early in interphase. In this model, condensin II may act as an ‘organizational bookmark’ by prioritizing intra-chromosomal folding immediately following mitotic exit. As condensin II is enriched at highly active regions of the genome marked by H3K4me3 [46, 60–62], its activity could potentially allow gene regulatory networks and chromatin compartments to favor *intra*- versus *inter*-chromosomal interactions. Further studies identifying the interactions driven by condensin II in relation to cohesin will be critical for understanding how these molecular machines cooperatively guide the genome through the cell cycle and development.

Finally, this report describes an efficient and scalable method of high-resolution chromosome painting using Oligopaint FISH technology [30]. Combined with a custom 3D-segmentation pipeline, quantitative measurements of chromosome size, shape, position, and overlap can be analyzed in a systematic and potentially high-throughput fashion [43, 63–66]. Moreover, the ability to conduct sequential rounds of hybridization with Oligopaints permits 3D analysis of many, if not all, CTs simultaneously. We anticipate this technology will lead to an enhanced ability to visualize and karyotype chromosomes in a number of systems, providing a novel battery of assays to better characterize how chromatin is packaged and spatially partitioned in the nucleus.

## Materials and methods

### Cell lines and tissue culture

Kc-167 (DGRC 1), S2 (DRSC 181), S2R+ (DGRC 150), CME L1 (DGRC 156), Clone 8 (CL.8+; DGRC 151), and BG3 (DGRC 166) cells were obtained from the *Drosophila* Genome Resource Center and were grown at 25°C following standard methods. Kc-167, S2, S2R+ cells were grown in sterile, filtered Schneider's medium (Invitrogen) supplemented with heat-inactivated fetal bovine serum (FBS; Hyclone SH30071.03) to a final concentration of 10% (v/v), and penicillin–streptomycin (50 units/mL penicillin, 50 μg/mL streptomycin; GIBCO). CME L1 and Cl.8+ cells were grown in Sang and Shields M3 insect media (Sigma) supplemented with 2% FBS and 5 μg/ml insulin plus 2.5% fly extract. BG3 cells were grown in M3, supplemented with 10% FBS and 10 μg/ml insulin. To ensure that experiments were done with log-phase cells, active cultures were split at a 1:5 ratio twice per week, and passaged at 2×10^6^ cells/mL 24 hours prior to experiments.

### Cell sorting

Approximately 10 million log-phase cells were harvested and pelleted by centrifugation (5 minutes at 1200rpm on table top centrifuge). Cells were then resuspended in 4% formaldehyde: 1% methanol, and fixed for 5 minutes at RT. Subsequently, cells were washed 2X in 1X PBS, followed by treatment with RNaseA (1 mg/mL in PBS) for 30 minutes at 37°. Cells were then washed twice in 1X PBS then incubated in a 2 μM solution of Propidium Iodide (PI) at RT for at least one hour, then strained with 4μm nylon mesh and resuspended to a final concentration of 5 million cells per mL. Fixed, stained cells were sorted on a Becton Dickinson FACSAria into G1, S, and G2 subpopulations. The cells were then settled on Poly-L-lysine-coated glass slides for 2 hours, where they were fixed again (4% formaldehyde for 5 minutes at RT), before being subjected to FISH. We confirmed that cells had been successfully sorted by assessing nuclear DNA content as determined by Hoechst staining (Fig 2H).

### RNAi-mediated knock down in cultured cells

T7 PCR was carried out using Taq DNA polymerase (Invitrogen), using the primers in S1 Table. dsRNA was generated using the MegaSCRIPT T7 kit (Applied Biosystems) and purified using the RNeasy Kit (Qiagen). Application of RNAi to cells was carried out by soaking in serum-free media according to published methods [67]. Briefly, for RNAi in a 6-well plate, 2x10^6^ cells were incubated with 20 μg of dsRNA in 1 mL of serum-free medium for 30 minutes. After incubation, 2 mL of serum-containing medium was added to cells, followed by incubation for 4 days. Control cells were treated with dsRNA targeting *brown*.

### Quantitative real-time PCR

For reverse transcription, total RNA was extracted from Kc167 cells 4 days after RNAi using the RNeasy kit (QIAGEN). Genomic DNA was removed from the sample with RNase-Free DNase (Promega). 2 μg RNA was used as the template for cDNA synthesis, which was performed using Maxima H minus RT (Thermo). The cDNA was then used as a template for quantitative real-time PCR, which was run using a BioRad iCycler iQ. The program was run in reaction volumes of 20 μl using EXPRESS SYBR GREEN SuperMix (Invitogen). Each qPCR reaction was run in triplicate. The following primers used for qPCR can be found in S2 Table.

### Immunofluorescence in cultured cells

Cells were settled on poly-L-lysine coated slides for 30 minutes, followed by fixation with 4% formaldehyde for 10 minutes. Slides were then washed three times (3X) in PBS-T (1X PBS with 0.1% Triton X-100) for 5 min with gentle rocking, and then blocked in 1% BSA in PBS-T, or 5% milk (w/v) in PBS-T for HP1a IF, for 30–60 minutes. 30 μl of blocking solution containing diluted primary antibodies was applied on the area of the slide containing fixed cells, covered with a 22x22 mm coverslip, and incubated in a humidified chamber overnight at 4°C. The next day, slides were washed 3X for 5 min in PBS-T, with gentle rocking, followed by incubation with 30 μl of secondary antibodies diluted in blocking solution for 1 hour at room temperature in a dark humid chamber. Slides were again washed 3X for 5 min in PBS-T, with gentle rocking, and were then washed with Hoechst (1:10,000 in PBS) for 5 minutes to visualize nuclei. Finally, slides were washed 2X in PBS-T for 5 minutes before mounting in SlowFade (Invitrogen). For IF/FISH, slides were subjected to IF as described here, followed by post-fixation with 4% formaledehyde for 10 minutes before proceeding with FISH as described below. Primary antibody dilutions were as follows: rabbit-anti PH3S10 (Millipore; 1:1000); mouse anti-alpha tubulin (Sigma; 1:50); chicken anti-CID (gift from Gary Karpen; 1:1000); mouse anti-H3K9me2 (abcam; 1:100), rabbit anti-H3K4me3 (abcam; 1:500), mouse anti-HP1a (C1A9, DSHB; 1:1000), rabbit anti-GFP (Invitrogen; 1:500). Secondary antibody dilutions were as follows: 488 goat anti-mouse (Jackson Labs, 1:100); Cy3 goat anti-rabbit (Jackson Labs, 1:200), 647 sheep anti-mouse (Jackson Labs, 1:100), 647 goat anti-chicken (Fisher, 1:500).

### Extraction of hemocytes from third-instar larvae

To extract hemocytes from third-instar wandering larvae, 10–12 larvae were dissected in 500 ul Schneider’s media. Heads were removed and all tissue was removed from the larval casing. The media and cells (minus tissue) was then harvested and pipetted through a 40um mesh filter, and hemocytes were concentrated by centrifugation at 600g for 5 minutes in table top centrifuge. Hemocytes were then washed 1X in PBS and spun onto poly-L-lysine coated slides using a cytocentrifuge (Shandon Cytospin 4; Thermo Fisher Scientific), and subsequently fixed in 4% PFA for 10 minutes. Slides were then washed 3X in PBS-T (0.1% Triton-X 100), before proceeding with FISH as described below.

### Generation of Oligopaint FISH probes

Oligopaint libraries were designed as previously described, using the Oligoarray 2.1 software [30, 32] and the DM3 genome build, and purchased from CustomArray. Chromosome paints were designed to have 42 bases of homology and a density of approximately 1 probe/kb. Cen, mid, and tel probes cover 4.28 Mb, 3.03 Mb, and 4.4 Mb, of chromosome 2L, respectively, and were designed with 42 bases of homology and densities of approximately 10 probes/kb. Coordinates for all paints can be found in S3 Table. Oligopaints were synthesized as previously described [68]. A detailed protocol can be found in S1 File.

### Oligopaint FISH on slides

Cells from log-phase cultures were settled on poly-L-lysine-treated glass slides for 30–60 minutes. Slides were subsequently fixed for 10 minutes with 4% formaldehyde in PBS-T (1X PBS with 0.1% Triton X-100) at room temperature (RT), followed by 3X 5 minute washes in PBS-T at RT, 1X 5 minute wash in 2×SSCT (0.3 m NaCl, 0.03 m sodium citrate, 0.1% Tween-20) at RT, and 1X 5 minute in 2×SSCT/50% formamide at RT. Slides were then pre-denatured in 2×SSCT/50% formamide at 92° for 2.5 minutes, then in 2×SSCT/50% formamide at 60° for 20 minutes. Primary Oligopaint probes in hybridization buffer (10% dextran sulfate/2xSSCT/50% formamide/4% polyvinylsulfonic acid (PVSA)) were then added to the slides, covered with a coverslip, and sealed with rubber cement. Slides were denatured on a heat block in a water bath set to 92° for 2.5 minutes, after which slides were transferred to a humidified chamber and incubated overnight at 37°. For whole-chromosome Oligopaints, 200pmol of probe was used per slide in a final volume of 25μl. For cen, mid, and tel Oligopaints, 20pmol of probe was used per slide in a final volume of 25μl. Approximately 16–18 hours later, coverslips were removed with a razor blade, and slides were washed in 2×SSCT at 60° for 15 minutes, 2×SSCT at RT for 15 minutes, and 0.2×SSC at RT for 5 minutes. For slides with whole chromosome Oligopaints, secondary probes (10pmol/25 μl) containing fluorophores were added to slides, again resuspended in hybridization buffer, and covered with a coverslip sealed with rubber cement. Slides were incubated at 37° for 2 hours in a humidified chamber, followed by washes in 2×SSCT at 60° for 15 minutes, 2×SSCT at RT for 15 minutes, and 0.2×SSC at RT for 5 minutes. All slides were washed with Hoescht DNA stain (1:10,000 in PBS) for 5 minutes, followed by 2X 5 minute washes in PBS before mounting in Slowfade (Invitrogen).

### 5-color FISH

To visualize all chromosomes in the same cells and for mitotic chromosome spreads, all primary oligos were designed with different secondary docking sites and hybridized at the same time. Secondary oligos labeling chromosomes X, 3L, and 3R were added and cells were imaged. Subsequently, unlabeled secondary probes with additional homology to the primary oligos were added to strip off the labeled secondary probes on 3L and 3R. Lastly, labeled secondary probes to 2L and 2R were added and cells were imaged again. Both sets of images were deconvolved independently using Leica autoquant LAS-X 3.3 software, and 2-D projections were merged and aligned to create images showing all 5 major chromosome arms.

### Metaphase chromosome spreads preparation

To induce mitotic arrest, 2 × 10^5^ cells were treated with 0.5 μg/ml demecolcine (Sigma-Aldrich) for 1 hour. Cells were then pelleted by centrifugation for 5 min at 600 *g* at room temperature and resuspended in hypotonic solution (250 ml of 0.5% sodium citrate), and incubated for 8 min. Following incubation, cells were placed in a cytofunnel and spun at 1,200 rpm for 5 min with high acceleration using a cytocentrifuge (Shandon Cytospin 4; Thermo Fisher Scientific). Cells were immediately fixed in 3:1 methanol: acetic acid for 10 min, followed by 3X 5 minute washes in PBS-T (PBS with 0.1% Triton X-100). After washes, slides were subjected to an ethanol row (3 minutes each in 70%, 90%, then 100% ethanol) at -20°. Slides were then dried at RT for 72 hours. Following drying, slides were denatured in 2xSSCT/70% formamide at 72° for 2.5 minutes, and again subjected again to an ethanol row at -20°. Subsequently, slides were dried for 10 minutes on a 42° heat block before performing FISH with Oligopaints as described above.

### Imaging, quantification, and data analysis

Images of cultured cells and hemocytes were acquired on a Leica wide-field fluorescence microscope, using a 1.4 NA 63x oil-immersion objective (Leica) and Andor iXon Ultra emCCD camera. All images were processed using the Leica LAS-X 3.3 software and exported as TIF files. Images were segmented and measured using a modified version of the TANGO 3D-segmentation plug-in for ImageJ [5]. Chromosome paints were segmented using either the ‘Hysteresis’ or ‘Spot Detector 3D’ algorithms. Statistical tests were performed using Prism 7 software by GraphPad. Figures were assembled in Adobe Illustrator. Underlying numerical data for all graphs are presented in S2 File and S3 File.

## Supporting information

S1 Fig Karyotype analysis of *Drosophila* cultured cell lines. (A-D) Left: Karyotype analysis of *Drosophila* cultured cells (A. BG3, B. Kc167, C. S2, D. S2R+). Representative chromosomes from the most frequent karyotype are shown. Scale bar equals 5 μm. Right: Heatmaps of interphase CT organization, showing pairwise contact frequencies and median CT overlap fraction.
(TIF)

S2 Fig CT organization in *Drosophila* cultured cell lines. (A) Oligopaints labeling chromosome X (green), 2L (red), and 2R (cyan) in 7 different *Drosophila* cell types. (B-C) Heatmaps of interphase organization, showing median CT overlap fractions between X, 2L, and 2R (B) or pairwise contact frequencies (C) from all cell lines shown in (A). Data shown represent one technical replicate (n≥300 cells). These data were confirmed by two additional technical replicates.
(TIF)

S3 Fig Radial positioning of *Drosophila* CTs across different cell lines. Radial position of chromosomes in nuclei from seven different Drosophila cell types, determined by shell analysis with five shells of equal volume, where shell 1 is closest to the nuclear periphery and 5 is the nuclear center. Data shown represent one technical replicate (n≥300 cells). These data were confirmed by two additional technical replicates.
(TIF)

S4 Fig Condensin II depletion in Kc167 cells. (A) qPCR confirming efficient knockdown of Cap-H2, Rad21, and Barren in Kc167 cells. Relative mRNA levels were normalized to levels in control RNAi samples and then to Act5c levels. Error bars were calculated across three different biological replicates. (B) IF/FISH in Kc167 cells depleted of Cap-H2, Barren, or Rad21. Heterochromatin is labeled with anti-H3K9me2 antibody (green), all chromosome Oligopaints are shown in magenta, and heterochromatin FISH probes (Het) labeling the AATAT, AATAG, AACAC, 359, and dodeca satellites in gray. Scale bar equals 5 μm. n>500 cells per condition. (C) Tukey box plot showing the mean and distribution (minus outliers) of the number of Het foci. Data shown are from a single biological replicate (n>500 cells each). These results were confirmed by two additional biological replicates, respectively. ***p < 0.0001; Mann-Whitney test. (D) Quantification of mitotic chromosome spreads performed after depletion of Brown (control) or Cap-H2 in Kc167 cells. 98% of control cells and 93% of Cap-H2 depleted cells showed the normal Kc167 karyotype (see S1 Fig; p = 0.33; Fisher’s Exact Test). (E) Scatter plot of nuclear volume (X-axis) versus 2L CT volume or X-2L overlap volume (Y-axis) of Cap-H2 depleted cells. Chromosome 2L volume data are shown in blue, while X-2L overlap data are shown in gray. R^2^ values were calculated in Prism. n = 534 cells. (F) qPCR confirming efficient knockdown of Cap-H2, Cap-D3, and SMC2 in Kc167 cells. Relative mRNA levels were normalized to levels in control RNAi samples and then to Act5c levels. (G) Oligopaints labeling chromosomes X (green), 2L (red), and 2R (cyan) on representative Kc167 cell nuclei depleted of Brown (control), Cap-D3, or SMC2. Dashed lines represent the nuclear edge. Scale bar equals 5 μm. (H) Tukey box plot showing CT volumes after depletion of Condensin II subunits. The data shown represent one biological replicate (n≥400 cells per RNAi). These data were confirmed by two additional biological replicates. (I) Bar graph showing average contact frequency between the X and 2L CTs after depletion of condensin II subunits. Error bars represent the standard deviation of three biological replicates (each n≥400 cells per RNAi). (J) Histogram showing X-2L CT overlap as a percent of X CT volume. Binned data from a single biological experiment are shown (n>400 cells per RNAi). These results were confirmed by two additional biological replicates. ***p ≤ 0.0001; Mann-Whitney test. (K) Average 2L radial position in the nucleus determined by shell analysis with five shells of equal volume, where shell 1 is closest to the nuclear periphery and 5 is the nuclear center. Averages from a single biological experiment are shown (n>400 cells per RNAi). These results were confirmed by two additional biological replicates. ***p < 0.0001; Mann-Whitney test.
(TIF)

S5 Fig Cap-H2 depletion in BG3 cells. (A) Oligopaints labeling chromosomes X (green), 2L (red), and 2R (cyan) on representative BG3 cell nuclei depleted of Brown (control), Cap-H2, or slmb. Dashed lines represent nuclear edge. Scale bar equals 5 μm. n≥350 cells per RNAi. (B) qPCR confirming efficient knockdown of Cap-H2 and SLMB in BG3 cells. Relative mRNA levels were normalized to levels in control RNAi samples and then to Act5c levels. (C) Tukey box plot showing CT volumes after depletion of Cap-H2 and slmb in BG3 cells. The data shown represent one biological replicate (n≥350 cells per RNAi). These data were confirmed by one additional biological replicate. (D) Bar graph showing average contact frequency between the X and 2L CTs (left) or X and 2R CTs (right) after depletion of Cap-H2 in BG3 cells. Error bars represent the standard deviation of two biological replicates (each n≥350 cells per RNAi). (E) Histogram showing X-2L CT overlap as a percent of X CT volume in BG3 cells depleted of Brown (control) or Cap-H2. Binned data from a single biological experiment are shown (n>350 cells per RNAi). These results were confirmed by one additional biological replicate. ***p < 0.0001; Mann-Whitney test. (F) Average 2L radial position in nuclei of BG3 cells depleted of Brown (control) or Cap-H2, determined by shell analysis with five shells of equal volume, where shell 1 is closest to the nuclear periphery and 5 is the nuclear center. Error bars represent the standard deviation of two biological replicates (each n≥350 cells per RNAi). ***p < 0.0001; Mann-Whitney test. (G) Heatmaps showing genome-wide contact frequencies for control and Cap-H2-depletd BG3 cells. The diagonal boxes are whole chromosome pairing frequencies. The data shown represent one biological experiment (n≥350 cells per RNAi). These results were confirmed by an additional biological replicate. (H) Heatmaps showing genome-wide CT overlap fractions for BG3 cells depleted of Brown (control) or Cap-H2. The data shown represent one biological experiment (n≥350 cells per RNAi). These results were confirmed by an additional biological replicate.
(TIF)

S6 Fig Cap-H2 overexpression and SLMB depletion. (A) Left: IF with anti-GFP antibody confirming expression of a copper-sulfate inducible Cap-H2-GFP construct. Uninduced cells were mock treated with H_2_O. Hoechst DNA stain is shown in blue. Scale bar equals 5 μm. Right: IF with anti-GFP and anti-H3K9me2 antibodies confirming knockdown of GFP-Cap-H2. Control cells were treated with Brown dsRNA. Hoechst DNA stain is shown in gray. Scale bar equals 5 μm. (B) Average 2L radial position in nuclei of Kc167 cells depleted of Brown (control) or SLMB, or transfected with pMT-CapH2-GFP and treated with water (uninduced) or CuSO_4_ (induced). The graph shows radial position determined by shell analysis with five shells of equal volume, where shell 1 is closest to the nuclear periphery and 5 is the nuclear center. Error bars represent the standard deviation of 3 biological replicates for SLMB RNAi (each n>350 cells per RNAi), or 3 technical replicates for over-expression. The changes shown are not significant (Mann-Whitney test). (C) Scatter plot of nuclear volume (X-axis) versus 2L CT volume or X-2L overlap volume (Y-axis) of Kc167 cells depleted of SLMB. Chromosome 2L volume data are shown in blue, while X-2L overlap data are shown in gray. R^2^ values were calculated in Prism. n = 656 cells. (D) qPCR confirming efficient knockdown of SLMB, CAL1, or HP1a in Kc167 cells. Relative mRNA levels were normalized to levels in control RNAi samples and then to Act5c levels. (E) IF with anti-CID anti-body confirming the depletion of CAL1. CAL1 is required to load CID protein at centromeres. DNA (Hoechst) is shown in blue, and CID is shown in white. Scale bar equals 5 μm. (F) IF with anti-HP1a antibody confirming the knockdown of HP1a protein. DNA (Hoechst) is shown in gray, HP1a in green, and H3K4me3 in red. Merged images show only HP1a and H3K4me3. Scale bar equals 5 μm.
(TIF)

S7 Fig Chromosome configurations are stable across cell cycle stages and cell types. (A) Schematic representation of the eight possible chromosome configurations with three-color FISH. The left four configurations were classified as open (cen-tel probes not touching), while the right four were classified as closed (cen-tel probes touching). (B) Bar graph showing the fraction of Kc167 (dark blue) or BG3 (light blue) cells in each of the eight configurations (n>500 cells per cell type). The differences seen are insignificant (Fisher’s exact test for open vs. closed configurations). (C) Bar graph showing the fraction of FACs sorted Kc167 cells in either G1 (light gray) or G2 (dark gray) in each of the eight configurations (n>300 cells per cell cycle phase). The differences seen are insignificant (Fisher’s exact test for open vs. closed configurations). (D) Bar graph showing the fraction of Kc167 cells in each of the eight configurations following either Cap-H2 or Rad21 depletion (n>500 cells per cell type). ***p < 0.0001; Fisher’s exact test (open vs. closed).
(TIF)

S1 Table T7 PCR primers for dsRNA synthesis. Primer names and sequences used for T7 PCR to generate dsRNA.
(DOCX)

S2 Table qPCR primers. Primer names and sequences used for qPCR.
(DOCX)

S3 Table Coordinates for Oligopaints. Genomic coordinates (Dm3) for chromosome-arm specific Oligopaints.
(DOCX)

S1 File Protocol for the preparation of Oligopaints. Detailed protocol for amplifying an Oligopaints library to generate FISH probes.
(PDF)

S2 File Underlying numerical data corresponding to the main figures. Spreadsheet containing all raw data used to generate graphs in the main figures
(XLSX)

S3 File Underlying numerical data corresponding to the supplemental figures. Spreadsheet containing all raw data used to generate graphs in the supplemental figures.
(XLSX)
