# Supplementary figures and images for: Condensin II drives large-scale folding and spatial partitioning of interphase chromosomes in Drosophila nuclei
Source: PLoS Genet. 2018 Jul 12;14(7):e1007393. doi: 10.1371/journal.pgen.1007393 (PMC6042687; doi:10.1371/journal.pgen.1007393)

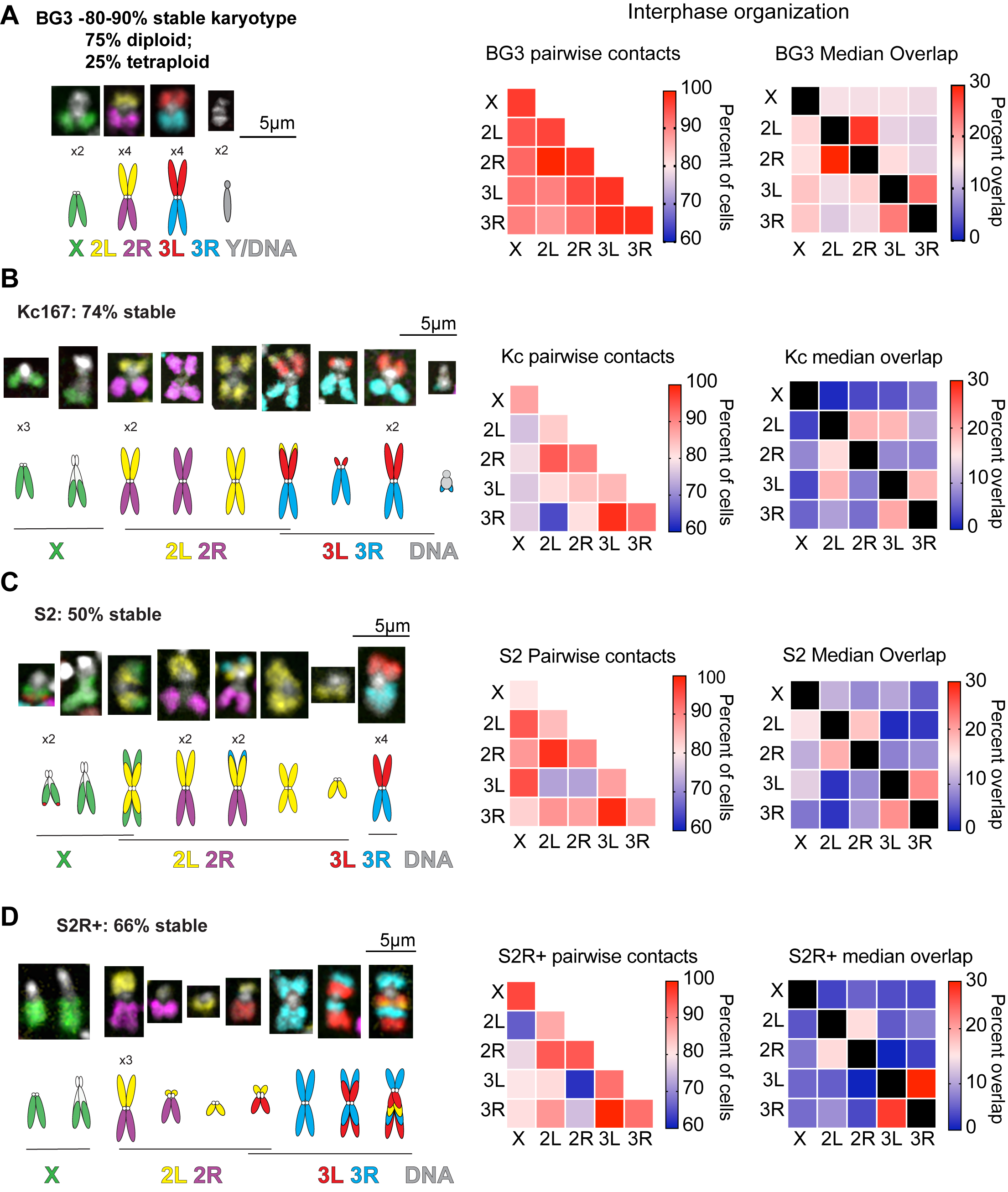

Supplement: S1 Fig — (A-D) Left: Karyotype analysis of Drosophila cultured cells (A. BG3, B. Kc167, C. S2, D. S2R+). Representative chromosomes from the most frequent karyotype are shown. Scale bar equals 5 μm. Right: Heatmaps of interphase CT organization, showing pairwise contact frequencies and median CT overlap fraction. (TIF) [file pgen.1007393.s001.tif]

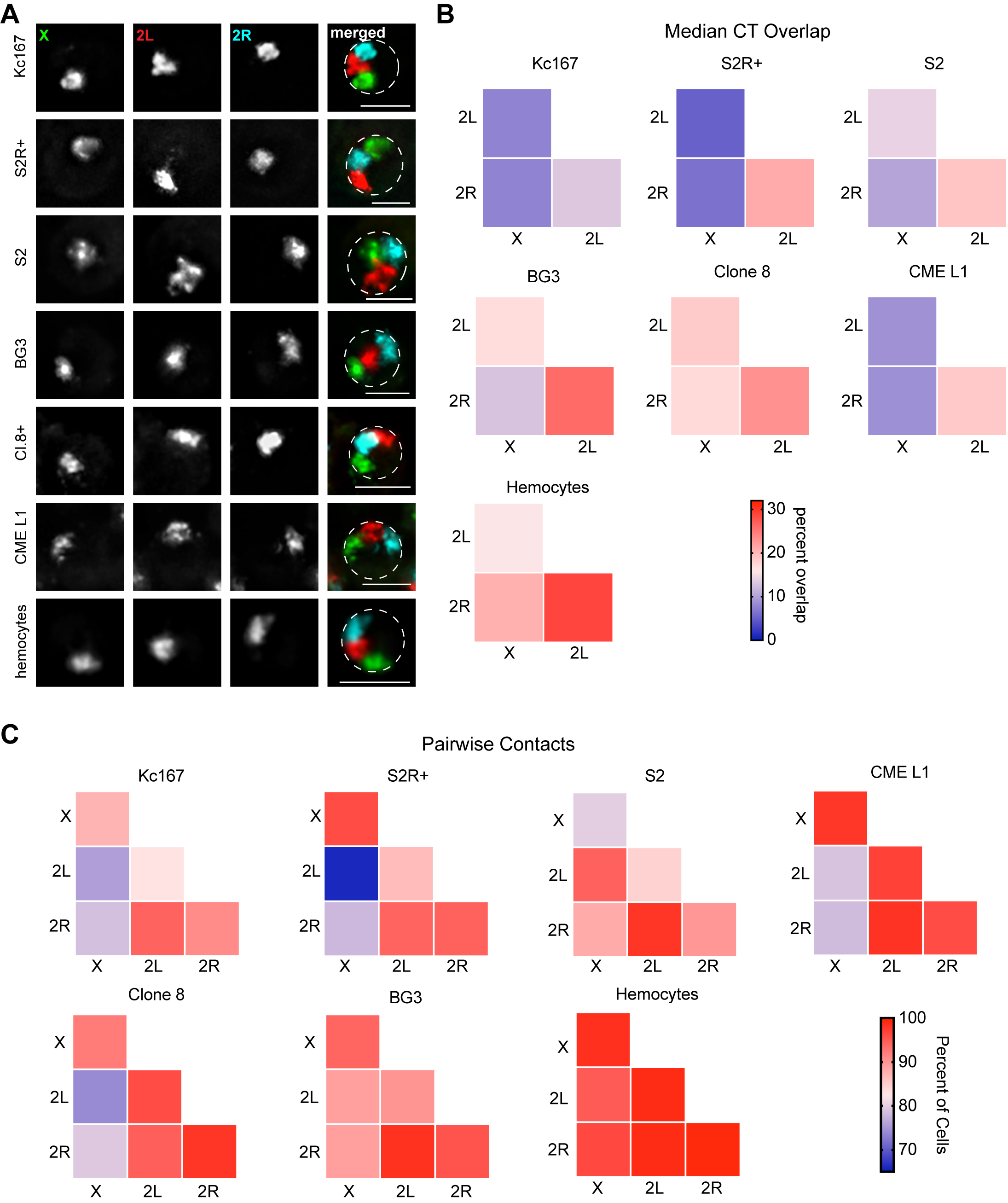

Supplement: S2 Fig — (A) Oligopaints labeling chromosome X (green), 2L (red), and 2R (cyan) in 7 different Drosophila cell types. (B-C) Heatmaps of interphase organization, showing median CT overlap fractions between X, 2L, and 2R (B) or pairwise contact frequencies (C) from all cell lines shown in (A). Data shown represent one technical replicate (n≥300 cells). These data were confirmed by two additional technical replicates. (TIF) [file pgen.1007393.s002.tif]

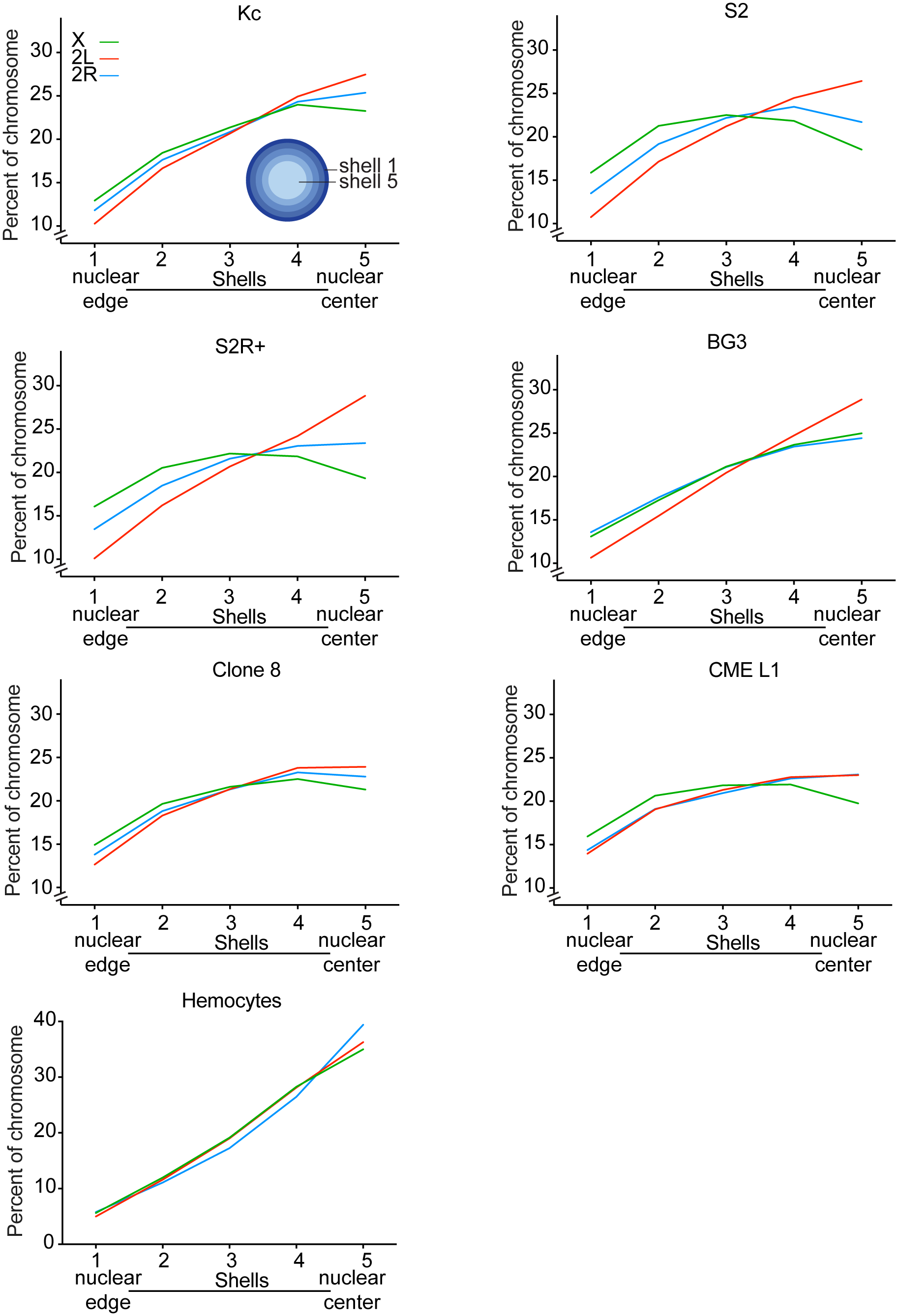

Supplement: S3 Fig — Radial position of chromosomes in nuclei from seven different Drosophila cell types, determined by shell analysis with five shells of equal volume, where shell 1 is closest to the nuclear periphery and 5 is the nuclear center. Data shown represent one technical replicate (n≥300 cells). These data were confirmed by two additional technical replicates. (TIF) [file pgen.1007393.s003.tif]

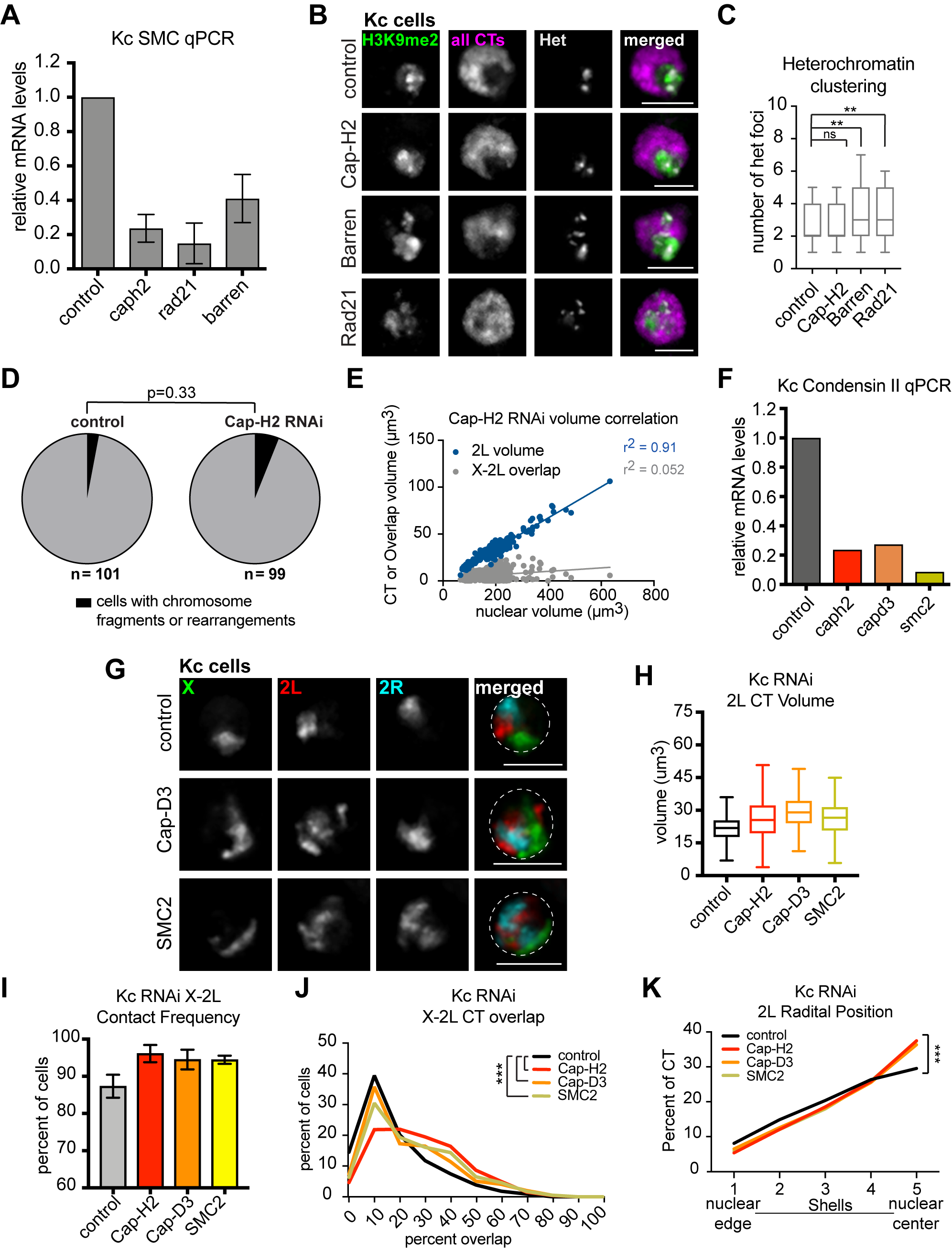

Supplement: S4 Fig — (A) qPCR confirming efficient knockdown of Cap-H2, Rad21, and Barren in Kc167 cells. Relative mRNA levels were normalized to levels in control RNAi samples and then to Act5c levels. Error bars were calculated across three different biological replicates. (B) IF/FISH in Kc167 cells depleted of Cap-H2, Barren, or Rad21. Heterochromatin is labeled with anti-H3K9me2 antibody (green), all chromosome Oligopaints are shown in magenta, and heterochromatin FISH probes (Het) labeling the AATAT, AATAG, AACAC, 359, and dodeca satellites in gray. Scale bar equals 5 μm. n>500 cells per condition. (C) Tukey box plot showing the mean and distribution (minus outliers) of the number of Het foci. Data shown are from a single biological replicate (n>500 cells each). These results were confirmed by two additional biological replicates, respectively. ***p < 0.0001; Mann-Whitney test. (D) Quantification of mitotic chromosome spreads performed after depletion of Brown (control) or Cap-H2 in Kc167 cells. 98% of control cells and 93% of Cap-H2 depleted cells showed the normal Kc167 karyotype (see S1 Fig; p = 0.33; Fisher’s Exact Test). (E) Scatter plot of nuclear volume (X-axis) versus 2L CT volume or X-2L overlap volume (Y-axis) of Cap-H2 depleted cells. Chromosome 2L volume data are shown in blue, while X-2L overlap data are shown in gray. R2 values were calculated in Prism. n = 534 cells. (F) qPCR confirming efficient knockdown of Cap-H2, Cap-D3, and SMC2 in Kc167 cells. Relative mRNA levels were normalized to levels in control RNAi samples and then to Act5c levels. (G) Oligopaints labeling chromosomes X (green), 2L (red), and 2R (cyan) on representative Kc167 cell nuclei depleted of Brown (control), Cap-D3, or SMC2. Dashed lines represent the nuclear edge. Scale bar equals 5 μm. (H) Tukey box plot showing CT volumes after depletion of Condensin II subunits. The data shown represent one biological replicate (n≥400 cells per RNAi). These data were confirmed by two additional biological rep [file pgen.1007393.s004.tif]

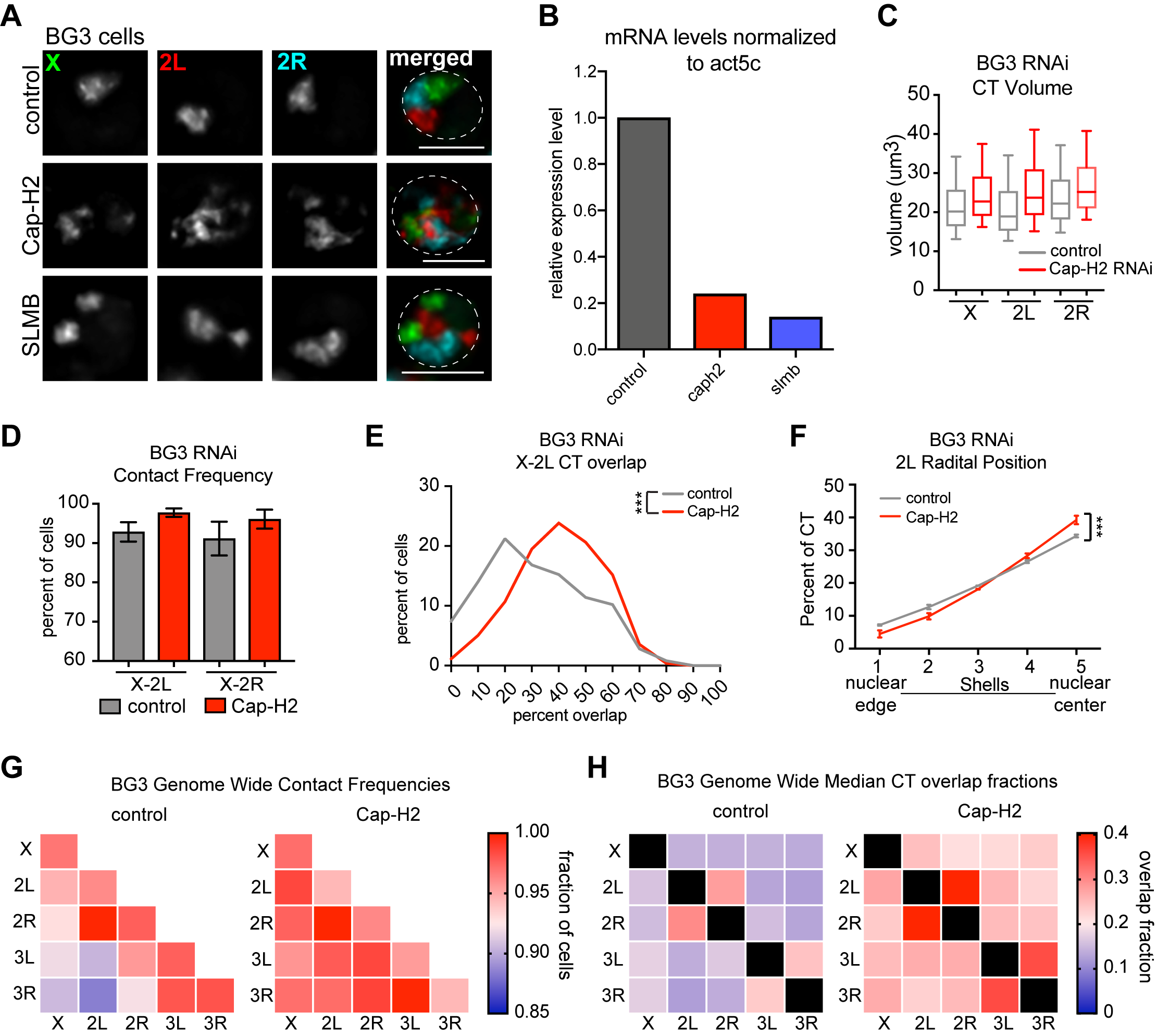

Supplement: S5 Fig — (A) Oligopaints labeling chromosomes X (green), 2L (red), and 2R (cyan) on representative BG3 cell nuclei depleted of Brown (control), Cap-H2, or slmb. Dashed lines represent nuclear edge. Scale bar equals 5 μm. n≥350 cells per RNAi. (B) qPCR confirming efficient knockdown of Cap-H2 and SLMB in BG3 cells. Relative mRNA levels were normalized to levels in control RNAi samples and then to Act5c levels. (C) Tukey box plot showing CT volumes after depletion of Cap-H2 and slmb in BG3 cells. The data shown represent one biological replicate (n≥350 cells per RNAi). These data were confirmed by one additional biological replicate. (D) Bar graph showing average contact frequency between the X and 2L CTs (left) or X and 2R CTs (right) after depletion of Cap-H2 in BG3 cells. Error bars represent the standard deviation of two biological replicates (each n≥350 cells per RNAi). (E) Histogram showing X-2L CT overlap as a percent of X CT volume in BG3 cells depleted of Brown (control) or Cap-H2. Binned data from a single biological experiment are shown (n>350 cells per RNAi). These results were confirmed by one additional biological replicate. ***p < 0.0001; Mann-Whitney test. (F) Average 2L radial position in nuclei of BG3 cells depleted of Brown (control) or Cap-H2, determined by shell analysis with five shells of equal volume, where shell 1 is closest to the nuclear periphery and 5 is the nuclear center. Error bars represent the standard deviation of two biological replicates (each n≥350 cells per RNAi). ***p < 0.0001; Mann-Whitney test. (G) Heatmaps showing genome-wide contact frequencies for control and Cap-H2-depletd BG3 cells. The diagonal boxes are whole chromosome pairing frequencies. The data shown represent one biological experiment (n≥350 cells per RNAi). These results were confirmed by an additional biological replicate. (H) Heatmaps showing genome-wide CT overlap fractions for BG3 cells depleted of Brown (control) or Cap-H2. The data shown represent one biological exp [file pgen.1007393.s005.tif]

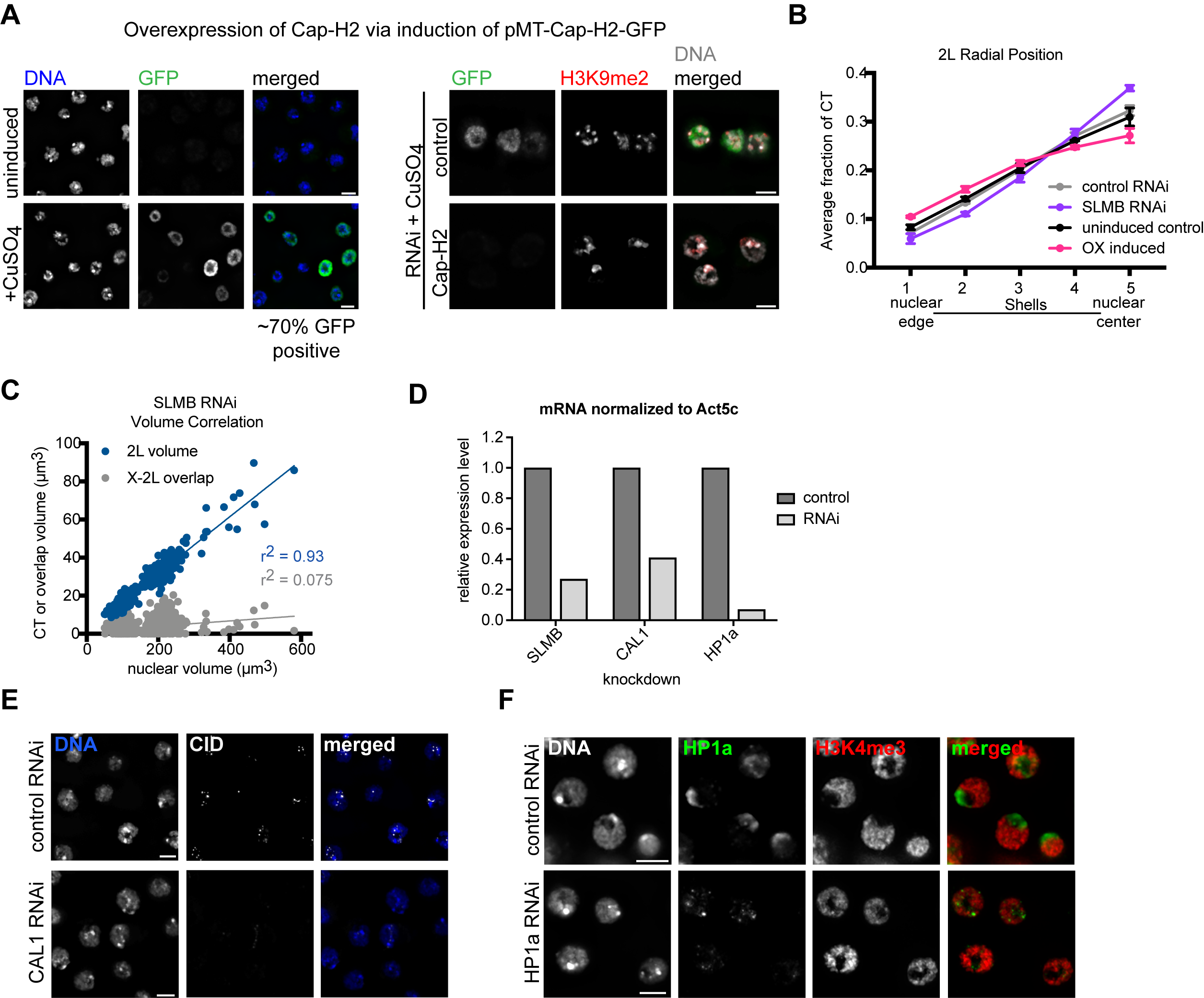

Supplement: S6 Fig — (A) Left: IF with anti-GFP antibody confirming expression of a copper-sulfate inducible Cap-H2-GFP construct. Uninduced cells were mock treated with H2O. Hoechst DNA stain is shown in blue. Scale bar equals 5 μm. Right: IF with anti-GFP and anti-H3K9me2 antibodies confirming knockdown of GFP-Cap-H2. Control cells were treated with Brown dsRNA. Hoechst DNA stain is shown in gray. Scale bar equals 5 μm. (B) Average 2L radial position in nuclei of Kc167 cells depleted of Brown (control) or SLMB, or transfected with pMT-CapH2-GFP and treated with water (uninduced) or CuSO4 (induced). The graph shows radial position determined by shell analysis with five shells of equal volume, where shell 1 is closest to the nuclear periphery and 5 is the nuclear center. Error bars represent the standard deviation of 3 biological replicates for SLMB RNAi (each n>350 cells per RNAi), or 3 technical replicates for over-expression. The changes shown are not significant (Mann-Whitney test). (C) Scatter plot of nuclear volume (X-axis) versus 2L CT volume or X-2L overlap volume (Y-axis) of Kc167 cells depleted of SLMB. Chromosome 2L volume data are shown in blue, while X-2L overlap data are shown in gray. R2 values were calculated in Prism. n = 656 cells. (D) qPCR confirming efficient knockdown of SLMB, CAL1, or HP1a in Kc167 cells. Relative mRNA levels were normalized to levels in control RNAi samples and then to Act5c levels. (E) IF with anti-CID anti-body confirming the depletion of CAL1. CAL1 is required to load CID protein at centromeres. DNA (Hoechst) is shown in blue, and CID is shown in white. Scale bar equals 5 μm. (F) IF with anti-HP1a antibody confirming the knockdown of HP1a protein. DNA (Hoechst) is shown in gray, HP1a in green, and H3K4me3 in red. Merged images show only HP1a and H3K4me3. Scale bar equals 5 μm. (TIF) [file pgen.1007393.s006.tif]

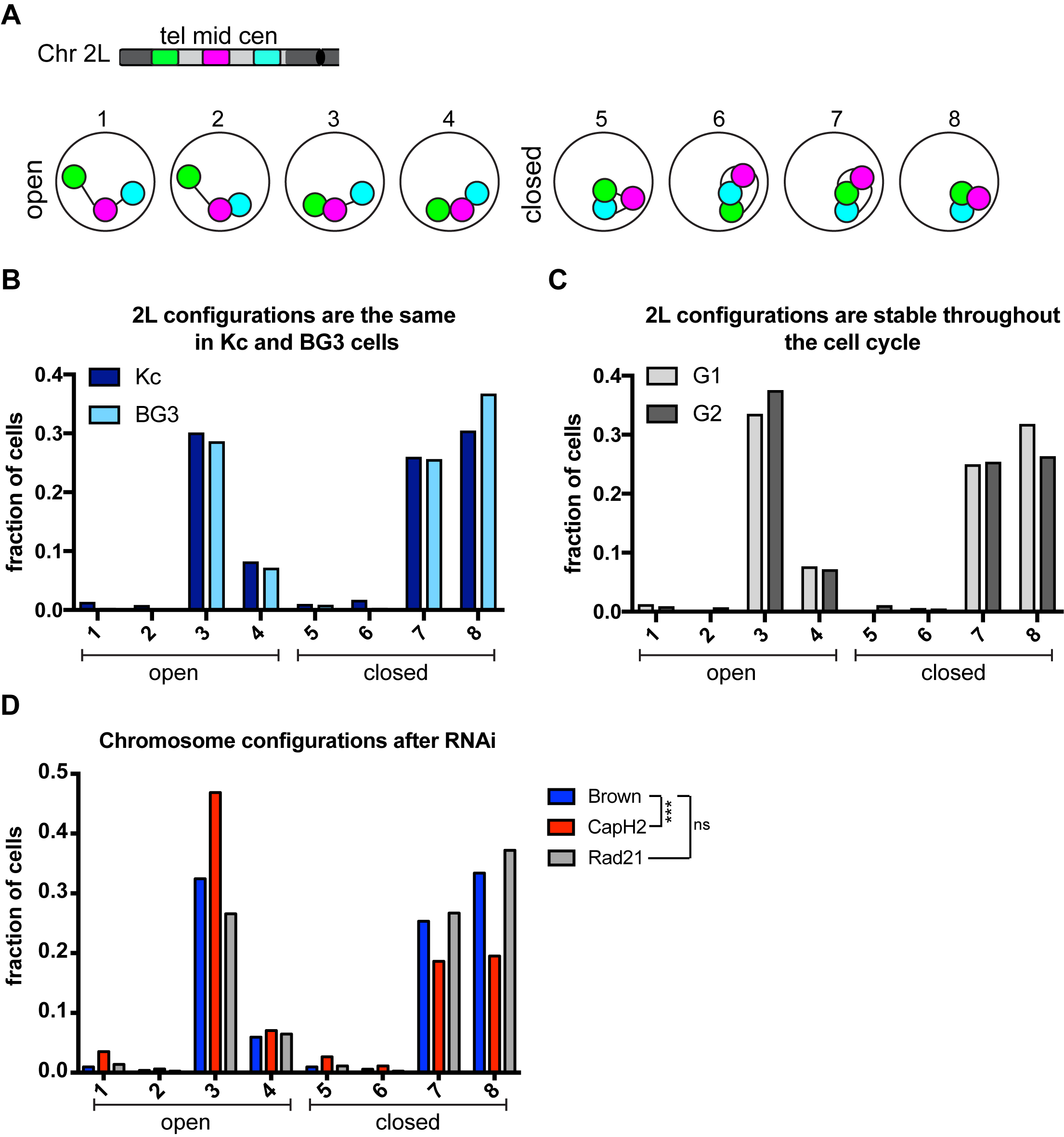

Supplement: S7 Fig — (A) Schematic representation of the eight possible chromosome configurations with three-color FISH. The left four configurations were classified as open (cen-tel probes not touching), while the right four were classified as closed (cen-tel probes touching). (B) Bar graph showing the fraction of Kc167 (dark blue) or BG3 (light blue) cells in each of the eight configurations (n>500 cells per cell type). The differences seen are insignificant (Fisher’s exact test for open vs. closed configurations). (C) Bar graph showing the fraction of FACs sorted Kc167 cells in either G1 (light gray) or G2 (dark gray) in each of the eight configurations (n>300 cells per cell cycle phase). The differences seen are insignificant (Fisher’s exact test for open vs. closed configurations). (D) Bar graph showing the fraction of Kc167 cells in each of the eight configurations following either Cap-H2 or Rad21 depletion (n>500 cells per cell type). ***p < 0.0001; Fisher’s exact test (open vs. closed). (TIF) [file pgen.1007393.s007.tif]
