# Supplementary material for: Condensin II drives large-scale folding and spatial partitioning of interphase chromosomes in Drosophila nuclei
Source: PLoS Genet. 2018 Jul 12;14(7):e1007393. doi: 10.1371/journal.pgen.1007393 (PMC6042687; doi:10.1371/journal.pgen.1007393)
Supplement: S1 Table — Primer names and sequences used for T7 PCR to generate dsRNA. (DOCX) [file pgen.1007393.s008.docx]

| **dsRNA Target** | **Primer 1** | **Primer 2** |
| --- | --- | --- |
| Brown | CTATGGCGTGACGTATATATTT | GATATTATCGATGTCGATCCAG |
| Cap-H2 | GAGCACATGACCACAAAGG | TATGCATTTGAATATCGGAAAG |
| Cap-D3 | AGAGGTGCGCGAGTTG | AGCCGCGCATAAACTCT |
| SMC2 | CTCCGCCAAGTATCTCAAGC | TGTTCTTGCAGTGTTGAGGC |
| Rad21 | GTATTGGAAAGAAACTGGAGGT | TCGTCACCCATTTCATGATTC |
| Barren | TGCCACATGCTGAAAGGTC | TTCTTCGCTCGTTGAGTG |
| Slmb | CACCAGGCGATCTCTGTA | ACACTGGATCGGTGCTGT |
| CAL1 | CTATAGGGATTGTTGATATCAGC | TGGATGCCAGGAAAGTTAGT |
| HP1a | TTAATCTTCATTATCAGAGTACCA | CCCTCTGGCAATAAATCAAAA |

Table S1. T7 PCR primers for RNA synthesis
