# Supplementary material for: Condensin II drives large-scale folding and spatial partitioning of interphase chromosomes in Drosophila nuclei
Source: PLoS Genet. 2018 Jul 12;14(7):e1007393. doi: 10.1371/journal.pgen.1007393 (PMC6042687; doi:10.1371/journal.pgen.1007393)
Supplement: S2 Table — Primer names and sequences used for qPCR. (DOCX) [file pgen.1007393.s009.docx]

Table S2. qPCR primers

| **Target** | **Primer 1** | **Primer 2** |
| --- | --- | --- |
| Act5c | AAGTTGCTGCTCTGGTTGTCG | GCCACACGCAGCTCATTGTAG |
| Cap-H2 | ACAGAACAGCCACGTTAGCC | CCAGGGAAGCCAGCATGTAG |
| Cap-D3 | AAAGCGAGGTGGAGTGGAG | CCAGGATGAGCACTTGACAC |
| SMC2 | GCTCCGCCTACCAGGAGTAT | GGGTCTCGCATTGCTTGAGAT |
| Rad21 | GGACCATGTCCCCGATTCAG | ATTCGCTCTCAGGACTTCCG |
| Barren | GAGACGATACCCTCGCATCC | TCGGGCAGCTTCTGATACAC |
| Slmb | AGCGGGAACTCTGCTTTCAG | TGTCCGTGTTGATAGTGGCAC |
| CAL1 | ACAACAGCGTCTACCTGGAG | GCCGTGTCGATGGACTGCAT |
| HP1a | AACGTACCACAGATGCGGAG | TGTCAGGCGGCCATTATTGT |
