# Supplementary material for: Condensin II drives large-scale folding and spatial partitioning of interphase chromosomes in Drosophila nuclei
Source: PLoS Genet. 2018 Jul 12;14(7):e1007393. doi: 10.1371/journal.pgen.1007393 (PMC6042687; doi:10.1371/journal.pgen.1007393)
Supplement: S3 Table — Genomic coordinates (Dm3) for chromosome-arm specific Oligopaints. (DOCX) [file pgen.1007393.s010.docx]

Table S3. Coordinates for Oligopaints

| **Target** | **Chromosome** | **Start** | **End** |
| --- | --- | --- | --- |
| X | X | 8603 | 22348002 |
| 2L | 2L | 5824 | 22767457 |
| 2R | 2R | 16362 | 20999406 |
| 3L | 3L | 21054 | 24399634 |
| 3R | 3R | 321 | 27799510 |
| tel | 2L | 5273 | 4402412 |
| mid | 2L | 9935314 | 12973080 |
| cen | 2L | 18496053 | 22780653 |
