# Supplementary material for: Condensin II drives large-scale folding and spatial partitioning of interphase chromosomes in Drosophila nuclei
Source: PLoS Genet. 2018 Jul 12;14(7):e1007393. doi: 10.1371/journal.pgen.1007393 (PMC6042687; doi:10.1371/journal.pgen.1007393)
Supplement: S1 File — Detailed protocol for amplifying an Oligopaints library to generate FISH probes. (PDF) [file pgen.1007393.s011.pdf]

Protocol - amplification of Oligopaints library and probe synthesis

Step 1: low cycle PCR reaction using raw library as template

<u>PCR amplification</u>
1 ul diluted raw library (100-200 pg/ul)
1.25 ul F primer (20 uM)
1.25 ul R primer (20 uM)
<u>21.5 ul H_2O</u>
50 ul total

Cycling:
1. 98°C - 3 min
2. 98°C - 5 s
3. 72 ^{o}C -15 s
4. Repeat 2-5 30x
5. 72 ^{o}C - 2 min
6. 12 ^{o}C - hold

<u>PCR clean-up (kit)</u>
- elute in 30ul
- dilute to 4ng/ul

Step 2: Adding T7 sites to Oligos

- Add **TAATACGACTCACTATAGGG** to the 5' end of the reverse primer

<u>T7 PCR (1X)</u>
2.5 ul 4ng/ul template
4 ul 10uM F primer
4 ul 10uM R primer
20 ul 5x buffer with $MgCl_2$ added (Invitrogen)
2 ul dNTPs (10mM)
0.5 ul taq polymerase (Invitrogen)
<u>H_2O to 100 ul</u>
100ul total

Cycling:
1. 94°C – 3 min
2. 94°C – 45 sec
3. 56°C – 30 sec
4. 72°C – 30 sec
5. Repeat 2-5 35X
6. 72°C – 5 min
7. 12°C - hold

<u>PCR clean-up (kit)</u>
- elute in 20ul

Step 3: *in vitro* transcription

<u>T7 RNA synthesis</u> (Hi-Scribe kit)
7 ul clean T7 PCR
2ul ATP
2ul CTP
2ul GTP
2ul UTP
2ul T7 buffer
2ul T7 pol mix
<u>1ul RNase OUT</u>
20 ul total
→ incubate at 37°C O/N

Step 4: conversion of RNA to RNA:DNA duplexes with reverse transcription

RT
20ul T7 RNA sample
7.5ul F sec primer (200uM)
9.6 ul dNTPs (100 mM dNTP mix, or 25 mM of each nucleotide)
30 ul 5x RT buffer
1.5ul RNase OUT
2ul Maxima RT-H
79.4ul H2O
150ul total
→ incubate at 50°C for 2 hours
heat water bath to 95°C for next step

Step 5: RNA degradation and DNA probe purification

Alkaline Hydrolysis
- Make 1:1 mixture of 0.5M EDTA:1M NaOH (need 1:1 ratio with sample [150 ul])
- degrade RNA by adding 150 ul EDTA:NaOH mix and incubating at 95°C for 10 minutes

Oligo Clean-up (Zymo-100 DNA Clean and Concentrator Kit)
- For each sample, make a 15mL conical tube containing:
    - 600 ul Oligo binding buffer (D4060-1-40, Zymo)
    - 2400 ul 100% ethanol
    - 300ul sample
- Load onto column and centrifuge or vacuum
- wash 2X with 2mL wash buffer
- spin column dry in microcentrifuge tube (~1min at max speed)
- elute in 150ul dH2O

→ nanodrop to get concentrations and convert to pM
